# Regulation of the bi-directional cross-talk between ovarian cancer cells and adipocytes by SPARC

**DOI:** 10.1038/s41388-019-0728-3

**Published:** 2019-02-14

**Authors:** Bincy John, Christine Naczki, Chirayu Patel, Alia Ghoneum, Shadi Qasem, Ziyan Salih, Neveen Said

**Affiliations:** 1 Department of Cancer Biology, Wake Forest University School of Medicine, Winston-Salem, NC USA; 2 Department of Pathology, Wake Forest University School of Medicine, Winston-Salem, NC USA; 3 Department of Urology, Wake Forest University School of Medicine, Winston-Salem, NC USA; 4 Present Address: Surgical Pathology, University of Kentucky College of Medicine, Lexington, KY USA; 5 Present Address: Department of Pathology and Laboratory Medicine, University of Louisville School of Medicine, Louisville, KY 40202 USA

**Keywords:** Ovarian cancer, Experimental organisms

## Abstract

Ovarian cancer (OvCa) exhibits a specific predilection for metastasis to the omentum. Our earlier studies highlighted the tumour-suppressor effect of secreted protein acidic and rich in cysteine (SPARC) in OvCa through multi-faceted roles inhibiting cancer cell interactions within the peritoneal milieu. The goal of this study is to investigate the role of SPARC in OvCa interactions with omental adipocytes and its role in OvCa colonization in the omentum. We employed multi-pronged approach using primary omental adipocytes from *Sparc* knockout mice, genetically engineered human omental adipocytes in 3D co-cultures with OvCa cells, as well as treatment with recombinant SPARC protein. We show that SPARC suppresses multistep cascade in OvCa omental metastasis. SPARC inhibited in vivo and adipocyte-induced homing, proliferation, and invasion of OvCa cells. SPARC suppressed metabolic programming of both adipocytes and OvCa cells and exerted an inhibitory effect of adipocyte differentiation and their phenotypic switch to cancer-associated phenotype. Mechanistic studies revealed that this effect is mediated through inhibition of cEBPβ-NFkB-AP-1 transcription machinery. These findings define a novel and functionally important role of SPARC in OvCa and not only bridge the knowledge gap but highlight the need to consider SPARC protein expression in therapeutic development.

## Introduction

Epithelial ovarian cancer (OvCa) is the leading cause of death from gynecologic malignancies in the United States [1]. More than 75% of patients are diagnosed at late stages with high mortality rates [2]. The main site of OvCa metastasis within the abdomen is the omentum, a vascular and adipocyte-rich tissue that drapes abdominal organs [2]. The role of omental adipocytes in promoting OvCa metastasis to the omentum has been recently established as they represent a significant source of factors that substantially promote OvCa-omental tropism, and colonization [3–8]. Adipocytes adjacent to cancer cells have been named cancer-associated adipocytes (CAAs) as they not only promote tumour growth, survival, and chemo-resistance [9], but they revert from mature, differentiated adipocytes to pre-adipocytes stage releasing their lipids to provide energy to cancer cells [3]. This phenotypic plasticity is controlled by factors that orchestrate differentiation, inflammation, and metabolic reprogramming in metabolic diseases, obesity, and cancer [10–15]. Strong evidence exists for a link between inflammation and adipocyte plasticity and their phenotypic switch to CAA; however, little is known about the signaling networks involved. Co-regulation and transactivation of CCAAT/enhancer-binding protein beta (C/EBPβ), Nuclear Factor-κB (NFκB) and activating protein-1 (AP-1) have been reported in the context of obesity, insulin resistance, and inflammation [16–19] as they are involved in the upregulation of inflammatory mediators [20, 21]. In addition, the three transcription factors (TFs) have been reported as crucial factors with transactivation circuitry in inflammation and cancer [22]. However, their pivotal role in OvCa-peritoneal dissemination is relatively unexplored.

Secreted protein acidic and rich in cysteine (SPARC), also termed osteonectin and BM-40, is an extracellular matrix (ECM) protein that exhibits contextual highly regulated expression in remodeling tissues to maintain tissue homeostasis (summarized in [23]). In this respect, SPARC has been shown to regulate the differentiation of mesenchymal, stem cells including adipocytes [24–26]. SPARC has been reported to inhibit adipogenesis as evidenced by the phenotype of *Sparc* null mice exhibiting osteoporosis and fatty bone marrow [24–26]. We have earlier reported that SPARC is an OvCa suppressor [5–8]. We reported that SPARC inhibited OvCa cell adhesion to various ECM proteins enriched in the peritoneal tumour microenvironment (TME) and peritoneal mesothelial cells [5–7]. SPARC exhibited an anti-proliferative effect that was attributed to inhibition of integrin- and growth factor-mediated survival signaling pathways [6–8]. We also reported that SPARC normalizes the TME through anti-inflammatory properties through suppression of the bi-directional cross-talk between cancer cells and macrophages and mesothelial cells [5–8, 27]. In addition, we reported that in the immunocompetent *Sparc* knockout mice (will be referred to as *SP*^–/–^), the enhanced peritoneal carcinomatosis was characterized by high levels and biological activity of pro-inflammatory mediators in tumours and ascitic fluid [6–8, 27]. These pro-inflammatory factors are reciprocated by cancer cells and stromal cells [7, 27, 28] and are correlated with advanced human disease, chemo-resistance, and poor prognosis [28]. Given the specific predilection of OvCa cells to the omentum and the reported inhibitory effects of SPARC on adipocyte differentiation [25, 29], we sought to investigate the role of SPARC in the bi-directional cross-talk between OvCa cells and omental adipocytes. We present evidence for the first time that the tumour-suppressor role of SPARC in OvCa is mediated through inhibition of OvCa cells–adipocytes interactions, the phenotypic plasticity of omental adipocytes, and metabolic programming.

## Results

### SPARC inhibits OvCa cell homing to the omentum in vivo and in vitro

**Fig. 1:**
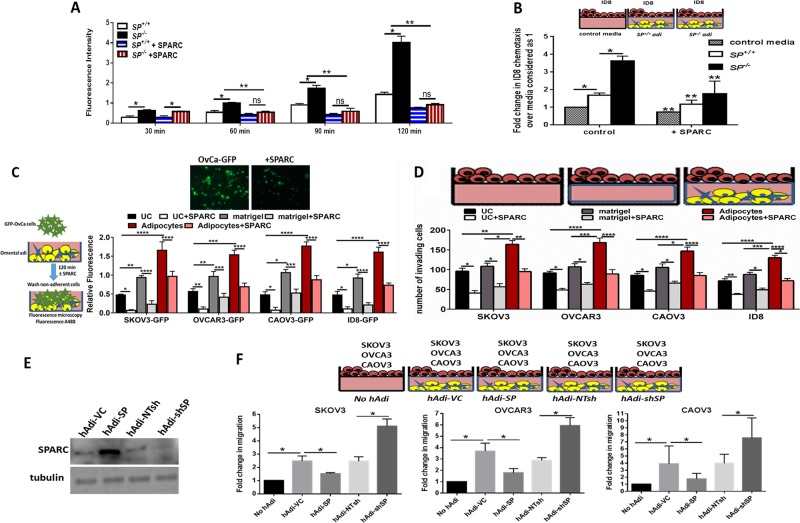
Effect of SPARC on homing of ovarian cancer (OvCa) cells to omental adipocytes. **a** In vivo homing of ID8-GFP cells to *SP*^*+/+*^ and *SP*^*–/–*^ omenta in the presence or absence of prior injection of 5 µg/ml SPARC. Bars represent means ± Standard error of the mean (SEM) of fluorescence intensity of adherent cells to omenta harvested at the indicated time points. *P < 0.05 comparing SP+/+ and SP–/–. **P < 0.05 comparing cells with and without SPARC treatment. ns, non-significant. **b** Schema of the in vitro homing/chemotaxis of ID8 cells towards *SP*^*+/+*^ and *SP*^*–/–*^ omental adipocytes. Bars represent means ± SEM of fluorescence intensity of ID8 cells that migrated through trans-wells towards adipocytes. Complete growth media were used as controls for migration (*n* = 4). *P < 0.05 comparing ID8 migration towards control media, SP+/+, SP–/– adipocytes in absence of SPARC. **P < 0.05 comparing migration of ID8 cells treated with SPARC with the corresponding condition in absence of SPARC treatment. **c** Schema of the adhesion assay of GFP-OvCa cells over-layed on top of adipocytes for 120 min (left). Bars represent means ± SEM of fluorescence intensity of adherent cells (*n* = 6/experimental condition). *p < 0.05 comparing ID8 migration towards control media, SP+/+,SP–/– adipocytes in absence of SPARC. **P < 0.05 comparing migration of ID8 cells treated with SPARC with the corresponding condition in absence of SPARC treatment. Photomicrographs of fluorescent adherent cells (top, 5×). **d** Schema of adipocyte-induced OvCa invasiveness through trans-well inserts towards omental adipocytes in the bottom chamber. Bars represent the means ± SEM of migrated cells counted in five random fields/insert, (*n* = 3). *P < 0.05 comparing ID8 migration towards control media, SP+/+, SP–/– adipocytes in absence of SPARC. **P < 0.05 comparing migration of ID8 cells treated with SPARC with the corresponding condition in absence of SPARC treatment. Student’s *t*-test. **e** Western blots showing the expression of SPARC after overexpression and knockdown in primary human omental adipocytes (hAdi). **f** Schema of the in vitro homing assay with GFP-labeled human OvCa cell lines. Bars represent means ± SEM of fold change of OvCa cells that migrated through trans-wells towards genetically engineered hAdi compared with cells migrated to control media (without adipocytes) considered as 1. (*n* = 4/experimental condition. Experiments were repeated three times). *p < 0.05 Student’s *t*-test with multiple comparisons

To determine whether SPARC inhibits ID8 cells preferential homing to omental adipocytes, we injected ID8-GFP cells intraperitoneally (ip) in *SP*^*–/–*^ and *SP*^*+/+*^ mice [5] and determined adherent ID8 cells harvested omenta (Fig. 1a) by measuring A_488_ fluorescence of green fluorescent protein (GFP)-labeled cells. We found that homing of ID8-GFP cells to *SP*^*–/–*^ omenta was significantly higher than to the *SP*^*+/+*^ at 60–120 min. To determine whether this increased homing was SPARC dependent, we injected recombinant murine SPARC (rSPARC 5 µg/100 µl phosphate buffered saline (PBS)) ip 30 min prior to ID8 injection. We found that SPARC inhibited ID8 homing to the omentum starting at 60 min post ID8 injection and mitigated the increased ID8-GFP adhesion to *SP*^*–/–*^ omenta (Fig. 1a). To clearly distinguish the role of omental adipocyte-SPARC, independent of other sources of SPARC in the complex peritoneal milieu, we constructed three-dimensional (3D) omental adipocyte culture composed of freshly isolated primary *SP*^*–/–*^ and *SP*^*+/+*^ omental adipocytes (Supplement Figure 1) embedded in reduced growth factor matrigel and co-cultured them with ID8-GFP cells as illustrated in Fig. 1b. We first determined the effect adipocyte*-*SPARC on ID8-GFP cell chemotaxis/migration or homing towards *SP*^*–/–*^ and *SP*^*+/+*^ omental adipocytes, and found that ID8 homing to *SP*^*–/–*^ omental adipocytes was significantly higher than to *SP*^*+/+*^ adipocytes (Fig. 1b). We next determined whether difference of homing of ID8 cells to adipocytes was mediated by differences in secreted factors and found that *SP*^*–/–*^ omental adipocytes exhibited significant increase in the levels of IL-6, CCL2/MCP1, CCL3/MIP1, VEGF, TNFα, IL-2, and leptin with modest though insignificant increase in levels of CTACK/CCL27, and TIMP1 (Supplement Figure. 2A). Neutralizing antibodies of the factors that exhibited significant differences between the two genotypes, significantly inhibited migration/homing of ID8 cells towards *SP*^*–/–*^ and *SP*^*+/+*^ omental adipocytes (Supplement Figure 2B). Of note that homing of ID8 cells to adipocytes isolated from mice bearing ID8 peritoneal tumours (will be referred to as CAA) was significantly higher than to normal adipocytes (normal Adi) isolated from non-tumour-bearing mice. Homing of ID8 to *SP*^*–/–*^ CAA was significantly higher than to *SP*^*+/+*^ CAA (Supplement Figure 2C). Furthermore, CAA exhibited significantly higher levels of the aforementioned inflammatory factors than normal adipocytes with *SP*^*–/–*^ CAA exhibiting significantly higher levels than *SP*^*+/+*^ CAA (Supplement Figure 2D). Adhesion of GFP-fluorescent human and murine OvCa cell lines SKOV3, OVCAR3, CAOV3, and ID8 (GFP-SKOV3, GFP-OVCAR3, GFP-CAOV3, and ID8-GFP) to omental adipocytes was inhibited by exogenous recombinant human and murine SPARC (rSPARC, Fig. 1c). Furthermore, rSPARC inhibited adipocyte-induced invasiveness human and murine OvCa cells (Fig. 1d). In addition, overexpression and depletion of SPARC in human adipocytes (hAdi; Fig. 1e) significantly inhibited/increased invasiveness of OvCa cells compared with their corresponding vector control adipocytes, respectively (Fig. 1f). Together these data highlight the paracrine effect of adipocyte-SPARC in inhibiting homing and invasiveness of OvCa cells through secreted inflammatory factors.

### SPARC inhibits adipocyte-induced OvCa cell proliferation in vitro and in vivo

**Fig. 2:**
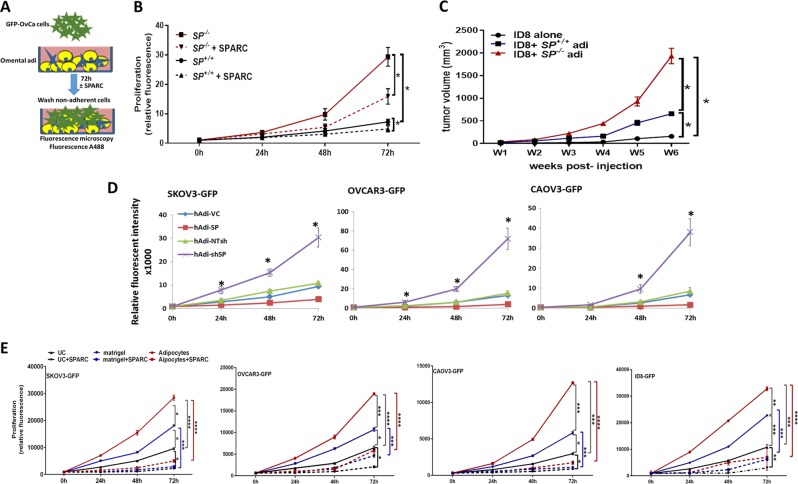
Effect of SPARC on omental adipocyte-induced OvCa cell proliferation. **a** Schema of the effect of human and murine omental adipocytes on GFP-OvCa cells proliferation. **b** Line graphs representing means ± SEM of changes in the proliferation (GFP fluorescence) of ID8-GFP cells over-layed on top of *SP*^*+/+*^ and *SP*^*–/–*^ adipocytes in the presence or absence of 5 µg/ml SPARC, over 72 h. **c** Means ± SEM of changes of tumours volumes after ID8 SC injection either alone or with *SP*^*+/+*^ and *SP*^*–/–*^ adipocytes (1:2) in athymic nude mice. *p < 0.05, between experimental conditions starting at week 3 post-injection (*n* = 8/group; two-way ANOVA with Tukey’s multiple comparison test). **d** Means ± SEM of changes in the proliferation (GFP fluorescence) of GFP-labeled human OvCa cells on top of genetically engineered human adipocytes over 72 h. (*n* = 6/experimental condition). *P < 0.05 comparing hAdi-SP and hAdi-shSP with their corresponding vector control at the indicated time points, two-way ANOVA. **e** Changes in the proliferation (GFP fluorescence) of OvCa cells over-layed on top of adipocytes in presence or absence of 5 µg/ml SPARC over 72h. (*n* = 6/experimental condition). *p < 0.05, **P < 0.001, ***p < 0.0001, comparing proliferation of GFP-cells on uncoated UC, wells (black), matrigel (blue) and adipocytes (red) in presence and absence of SPARC, two-way ANOVA with Tuckey's multiple comparison test

To further investigate the adipocyte-SPARC on OvCa cell proliferation, we incubated ID8-GFP cells in direct contact with *SP*^*–/–*^ and *SP*^*+/+*^ omental adipocytes and found that ID8 proliferation was significantly higher (~3-folds) compared with those incubated with the *SP*^*+/+*^ as determined by measuring the GFP fluorescence over 72 h. This effect was partially mitigated by treating co-cultures by rSPARC (Fig. 2a, b). Similar results were obtained by parallel experiments in which OvCa cells were separated from adipocytes by 0.4 µm trans-well inserts placed in direct contact with the differentiated adipocytes for 72 h. Adipocytes and OvCa cells were collected, trypsinized, and counted by Trypan blue exclusion at the same time points. Consistent results were observed with OvCa counting and measuring fluorescent intensity of GFP, whereas the number of viable adipocyte did not exhibit significant change during the experiment (data not shown). To further confirm the role of adipocyte-SPARC on ovarian tumour growth in vivo, we injected ID8 cells with *SP*^*+/+*^ and *SP*^*–/–*^ omental adipocytes (1:2, cancer cell:adipocyte ratio) subcutaneously in 6-week-old female athymic nude mice. We found that ID8 cells injected with *SP*^*–/–*^ adipocytes produced ~3.5 times larger tumours than those injected with *SP*^*+/+*^ adipocytes (Fig. 2c). Similar effect of adipocyte-SPARC was observed with early passage differentiated primary human omental (pre)adipocytes genetically manipulated SPARC expression (by overexpression and depletion, respectively) incubated with SKOV3-GFP, OVCAR3-GFP, and CAOV3-GFP (Fig. 2d). Furthermore, exogenous rSPARC inhibited adipocyte-induced OvCa cell proliferation (Fig. 2e). It is noteworthy that OvCa cell proliferation was significantly higher when plated on adipocyte/matrigel plugs compared with plating on matrigel alone or on uncoated wells; an effect that was inhibited by rSPARC (Fig. 2e). These results further support the paracrine effect of adipocyte-SPARC and rSPARC inhibiting adipocyte-induced OvCa cell proliferation, homing, adhesion, and invasiveness.

### Effect of SPARC on co-culture induced inflammatory chemokines in OvCa cells and adipocytes

**Fig. 3:**
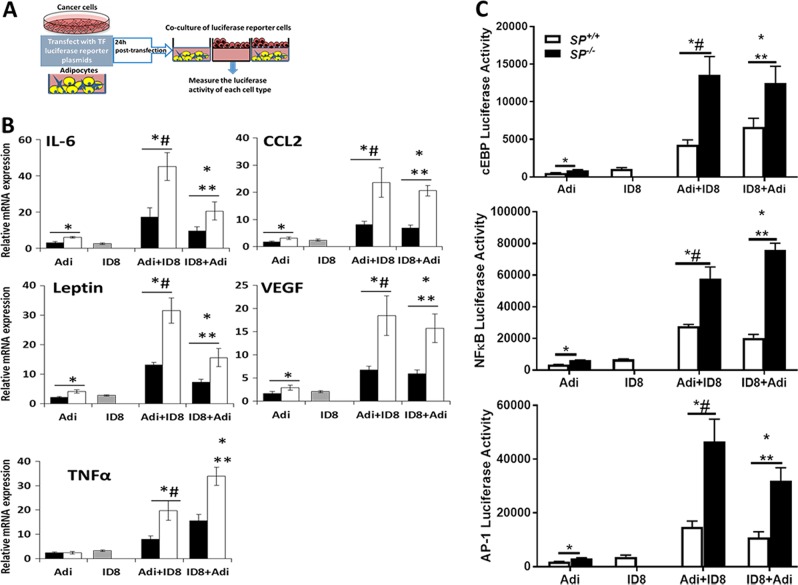
Adipocyte-SPARC suppresses the reciprocal transcriptional activity of pro-inflammatory/adipogenic factors. **a** Schematic illustration of the experimental design and the trans-well assays for the single and co-cultures. **b** The levels of adipokines in *SP*^*+/+*^ and *SP*^*–/–*^ omental adipocytes and ID8 cells in single and in co-cultures for 24 h was determined by qRT-PCR. **c** Transcriptional activity of cEBP, NFκB, and AP-1 in primary *SP*^*+/+*^ and *SP*^*–/–*^ omental adipocytes and ID8 cells in single and in co-cultures was determined by measuring the luciferase reporter activity in each cell type. Results were normalized to fold change of DNA content of each cell type measured before and after the experiment as determined by CyQuant assay. Bars represent mean ± SEM from one of three experiments, performed in triplicate. *p < 0.05 Student’s *t-*test comparing *SP*^*+/+*^ and *SP*^*–/–*^ adipocytes; ^#^*p* < 0.05 Student’s *t-*test comparing adipocytes in single to co-cultures with ID8 cells; and **p < 0.05 Student’s *t-*test comparing ID8 cells in single to co-culture with *SP*^*+/+*^ and *SP*^*–/–*^ adipocytes

To further determine the effect of adipocyte-SPARC on the expression of cytokines in both tumours cells and adipocytes, we determined the mRNA expression of cytokines and chemokines in ID8 and *SP*^*–/–*^ and *SP*^*+/+*^ adipocytes in mono- and co-cultures. We found that the association of ID8 cells with adipocytes significantly upregulated the expression of IL-6, CCL2, TNFα, VEGF, and leptin transcripts in both cell types compared with mono-cultures (Fig. 3a, b). The expression of the transcripts of the aforementioned adipokines were significantly higher in *SP*^*–/–*^ adipocytes than in *SP*^*+/+*^ (1.9-, 1.8-, 2-, 2.5-, and 1.7-fold for IL-6, CCL2, VEGF, TNFα, and leptin, respectively). Co-culture with ID8 cells significantly upregulated the expression of each factor in *SP*^*–/–*^ adipocytes over *SP*^*+/+*^ by 2.6- to 3-folds. Concomitantly, ID8 cells co-cultured with *SP*^*–/–*^ adipocytes exhibited ~2- to 2.7-fold induction of these transcripts, compared with co-culture with *SP*^*+/+*^ adipocytes (Fig. 3a, b).

In silico analysis of the common transcriptional regulation of the aforementioned chemokines using (http://opossum.cisreg.ca/cgi-bin/oPOSSUM3/), predicted common transcription machinery regulated by cEBPβ, NFκB, and AP-1. These TFs are not only implicated in adipocyte differentiation but are also recognized as oncogenes and markers of inflammation and aggressiveness of many cancers including OvCa [21, 30–34]. To monitor the concomitant changes in OvCa cells and adipocytes in co-culture, we co-cultured ID8 cells on 0.4 µm inserts on top of 3D *SP*^*–/–*^ and *SP*^*+/+*^ omental adipocytes and determined the changes in NFkB, AP-1, and cEBPβ promoter activity in both cell types using luciferase reporters [35, 36]. Co-culture of *SP*^*–/–*^ and *SP*^*+/+*^ adipocytes with ID8 cells elicited profound significant increase in the activity of these TFs over single-cell culture, with *SP*^*–/–*^ adipocytes exhibiting significantly higher levels than the *SP*^*+/+*^. ID8 co-cultured with *SP*^*–/–*^ adipocytes exhibited significantly higher luciferase reporter activation than those co-cultured with *SP*^*+/+*^ adipocytes (Fig. 3c). Consistently, human OvCa cell lines co-cultured with omental adipocytes depleted of SPARC (Supplement Figure 3A) exhibited significant increase in the activation of the three TFs in co-cultures compared with those in mono-cultures. Conversely, overexpression of SPARC in adipocytes, inhibited the activation of the three TFs in both OvCa cells and adipocytes (Supplement Figure 3B). Of note is that genetic manipulation of SPARC in primary hAdi by overexpression and knockdown significantly inhibited/increased the luciferase activity of NFκB and AP-1 but not cEBP prompter reporters in mono-cultures, respectively. Importantly, treating OvCa cells and adipocytes in single and co-culture with rSPARC phenocopied the effect of SPARC overexpression in adipocytes and significantly inhibited the promoter activity of the three TFs in OvCa cells and adipocytes (Supplement Figure 3B). It is noteworthy that the significantly reciprocated increase in promoter activity of the three TFs reported above is due to increase in activation and not due to increased cell proliferation as we determined changes cell proliferation of in mono- and co-cultures before (0 h) and after 24 h (end of the assay), and normalized the luciferase reporter activity in each cell type to the fold change in cell proliferation during the experiment. Differentiated human and murine adipocytes did not exhibit changes in proliferation as determined by CyQuant assay (data not shown). However, monoculture of ID8 cells exhibited 1.5-fold increase in proliferation, whereas, in co-culture with *SP*^*+/+*^ and *SP*^*–/–*^ adipocytes, ID8 exhibited 2- and 3.8-fold change in proliferation (Supplement Figure 4A). Similarly, mono-cultures of SKOV3, OVCAR3, and CAOV3 exhibited 1.3-, 1.2-, and 1.7-fold change in proliferation in 24 h, respectively; whereas, in co-cultures with hAdi depleted, the three cell lines exhibited ~2- to 2.5-fold increase proliferation (Supplement Figure 4B, C). OvCa cells in co-cultures, with adipocytes overexpressing SPARC exhibited 1.3- to 1.4-fold increase in their numbers after 24 h; whereas co-cultures with adipocytes depleted of SPARC exhibited 3- to 4-fold increase in cell proliferation (Supplement Figure 4B, C).

Consistently, treatment of OvCa cells and primary omental adipocytes in mono-and co-cultures with rSPARC downregulated the transcript levels of *IL-6, CCL2, VEGFA, TNFα*, and *LEP*, all are downstream target genes of the three TFs in both OvCa cells and hAdi (Supplement Figure 5A, B).

### Effect of SPARC on the activation of NFkB, AP-1, and cEBPβ in OvCa cells and adipocytes in vivo

**Fig. 4:**
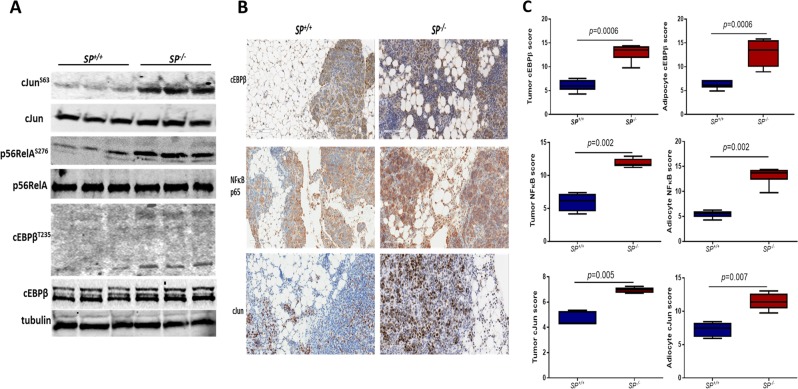
SPARC suppresses pro-inflammatory/adipogenic factors in ID8 intraperitoneal tumour. **a** Immunoblots of total and phosphorylated cJun, p65NFκB, in lysates from ID8 intraperitoneal tumours growing in *SP*^*–/–*^ and *SP*^*+/+*^ mice. Tubulin was used as a loading control. **b** Immunostaining of cEBPβ, NFκB, and cJun in tumours dissected from *SP*^*+/+*^ and *SP*^*–/–*^ (magnification, ×200). **c** Box plots of the expression scores of the nuclear transcription factors in tumour cells and adipocytes of both genotypes. *p* < 0.05, Mann–Whitney test

**Fig. 5:**
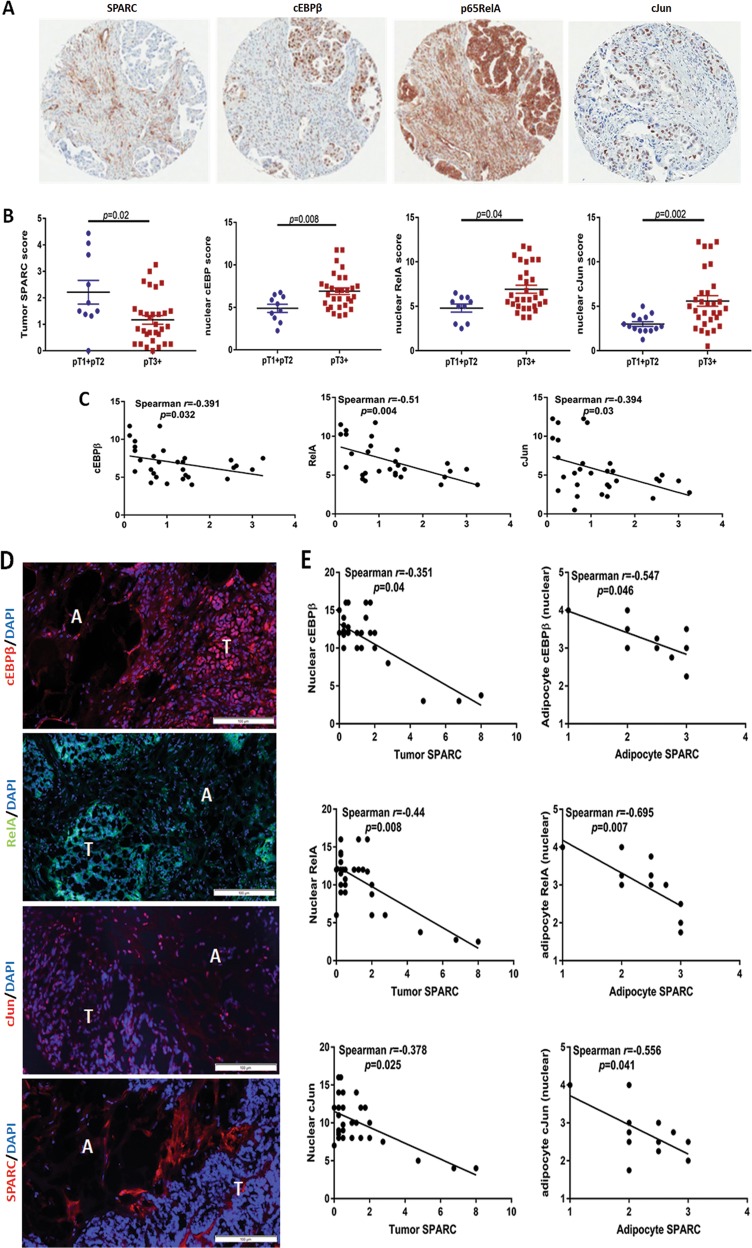
Correlation of tumour SPARC and cEBPβ, NFkB, and AP-1. **a** Photomicrograph of stage III HGSC specimens (CHTN) showing distinctive compartmentalization of SPARC in the cancerous vs stromal compartments and the expression of cEBPβ, NFkB, and cJun. **b** Scatter plots of the expression scores of SPARC, cEBPβ, NFkB, and cJun. *p* < 0.05, Mann–Whitney test. **c** Spearman’s correlation of the expression scores of tumour-SPARC and nuclear transcription factors. **d** Immunofluorescence staining of the expression of omental metastases of HGSC specimens, showing the expression of cEBPβ, RelA, cJun, and SPARC in the tumour (T)–adipocyte (A) interface. **e** Spearman’s correlation of the nuclear expression of tumour and adipocyte cEBPβ, RelA, and cJun with tumour and adipocyte SPARC. Scale bars 100 µm

To determine the effect of SPARC on the three TFs in OvCa-adipocytes interactions in vivo, we first determined the levels of total and phosphorylated proteins in lysates of syngeneic ID8 omental tumours that developed after ip injection in *SP*^*–/–*^ and *SP*^*+/+*^ mice. We found marked increase in phosphorylation of cJun (S^73^), p65RelA (S^276^), as well as cEBPβ (T^235^) in *SP*^*–/–*^ tumour lysates compared with *SP*^*+/+*^ tumours (Fig. 4a). Immunostaining of ID8 omental tumours revealed significant increase in frequency of nuclear p65RelA, cJun and cEBPβ in both tumour cells and the juxtra-tumoral adipocytes in *SP*^*–/–*^ tumours compared with the *SP*^*+/+*^ tumours (Fig. 4b, c). Importantly, immunostaining of human OvCa specimens from OvCa tissue microarray (TMA, Fig. 5a, b) revealed significant increase in the expression of nuclear TFs in advanced stage tumours, T3 and T4 (referred to thereafter as T3+), compared with early stage tumors, T1 and T2 (referred to thereafter as T1+T2, Fig. 5a, b). Importantly, we found significant negative correlation between tumour SPARC with nuclear expression of the p65RelA, cJun, and cEBPβ in advanced stage (T3+) OvCa specimens (Fig. 5c) in TMAs where only tumour cores are present. In an independent cohort of stage T3+ human OvCa specimens with adjacent omental tissue, we found significant decrease of SPARC expression in the cancerous compartment with distinctive expression in the stroma (Fig. 5d). In this cohort, SPARC expression in tumour cells negatively correlated with the nuclear expression of the three TFs. In addition, SPARC expression in the juxtra-tumoral adipocytes negatively correlated with nuclear expression of the TFs in the adipocytes (Fig. 5d, e). These data further confirm the negative correlation between SPARC protein expression and the activation of these TFs in tumour cells and adipocytes. It is noteworthy that in the human tissues, adipocytes exhibit detectable expression levels of SPARC protein with increasing intensity of expression as the juxta-tumoral adipocyte become smaller in size (Supplement Figure 6). Together with our earlier reports that SPARC is required for stromal cell differentiation and acquisition of cancer-associated phenotype [36], we sought to determine the effect of SPARC on adipocyte differentiation and acquisition of CAA phenotype.

### Effects of SPARC on reciprocal cross-talk between adipocytes and OvCa cells

**Fig. 6:**
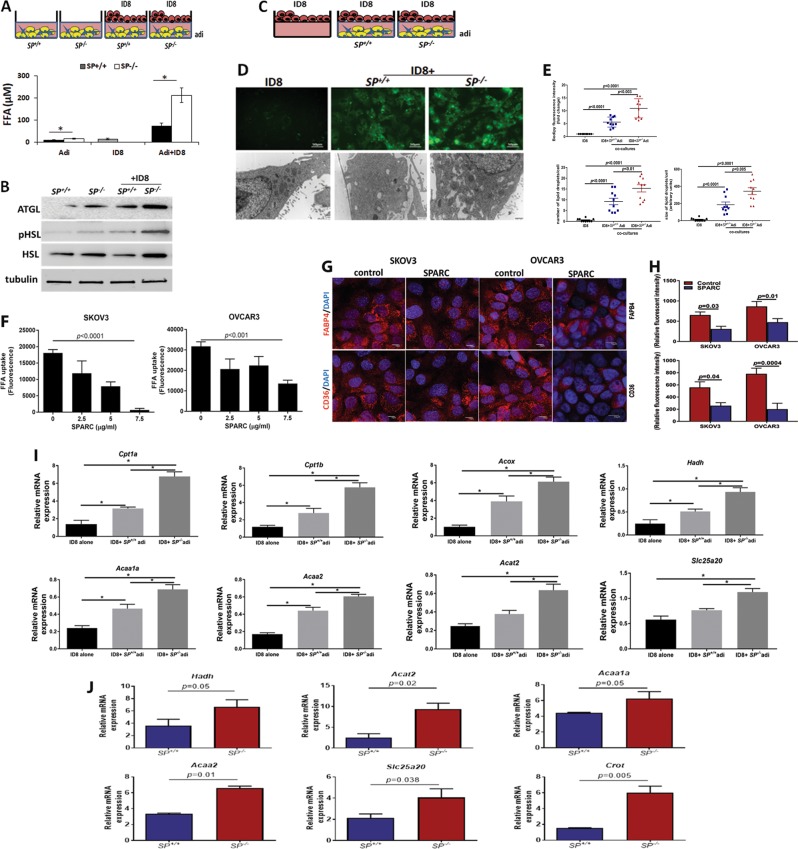
Effect of SPARC on metabolic programming of adipocytes and OvCa cells. **a** Schema of the co-culture (upper), bars represent means ± SEM of FFA release in media of co-cultures for 48h. *p < 0.05, Student’s *t*-test. **b** WB showing increased lipases in adipocytes in mono- and co-cultures with ID8 cells. **c** Schema showing co-cultures of ID8 and adipocytes. **d** Photomicrographs of FA uptake by ID8 cells visualized by Bodipy staining of in co-cultures (upper, 40×; scale bar 50 µm) and electron microscopy (lower, 6800×; scale bar, 500 nm). **e** Quantification of Bodipy fluorescence intensity in the in ID8 cells in mono- and co-cultures with adipocytes. The number and size of lipid droplets (lower) was quantified in 10 cells/condition. Plots represent means ± SEM of the fluorescent intensity quantified in 10 fields/experimental condition (*n* = 3 experiments), and unpaired *P* < 0.05, *t*-test. **f** Effect of SPARC on FA uptake by human OvCa cells. *p* < 0.05, one-way ANOVA. **g** Immunofluorescence showing the effect of SPARC (5 µg/ml) on FABP4 and CD36 expression in SKOV3 and OVCAR3 cells. Scale bars, 10 µm. **h** Bars report means ± SEM of fluorescence intensity of FABP4 and CD36 quantified in 10 fields/experimental condition, Student’s *t*-test. **i** mRNA expression of enzymes involved in FA oxidation of ID8 in mono- and co-culture with *SP*^*+/+*^ and *SP*^*–/–*^ adipocytes. Bars report means ± SEM of a representative of three independent experiments each in triplicate. *p < 0.05. Student’s *t*-test. **j** Bars depict mean ± SEM of the changes in the transcript levels (*n* = 3/genotype). *p-*values are determined by unpaired Student’s *t*-test

Earlier reports demonstrated that tumour-adjacent adipocytes undergo phenotypic changes into CAAs to support cancer cells growth and survival. In addition to expression of pro-inflammatory cytokines and TFs [37], CAA release their lipids through lipolysis [3, 4]. Using the heterotypic cultures of ID8 and adipocytes, we found increased free fatty acids (FFA) production in conditioned media (CM) of co-cultures compared with single-cell cultures with higher FFA of in co-cultures including *SP*^*–/–*^ adipocytes (Fig. 6a). This effect was inhibited by treating co-cultures of ID8 cells with *SP*^*+/+*^ and *SP*^*–/–*^ adipocytes with inhibitors of NFkB, AP-1, and cEBPβ (Supplement Figure 7) inhibited co-culture-induced fatty acid (FA) release from adipocytes with modest effect on the adipocytes in monoculture (Supplement Figure 8). Consistently, *SP*^*–/–*^ adipocytes expressed higher levels of adipose triglyceride lipase (ATGL) and total and phospho-hormone-sensitive lipase (HSL), the rate-limiting enzymes in the breakdown lipids and mobilization of FFA from adipocytes [38], compared with *SP*^*+/+*^ adipocytes (Fig. 6b). ID8 cells exposed to the same co-culture conditions with adipocytes exhibited higher levels of intracellular FA and larger lipid droplets in co-cultures with *SP*^*–/–*^ compared with *SP*^*+/+*^ adipocytes as determined by Bodipy staining and electron microscopy (Fig. 6c–e). To further confirm whether these observations were due to a direct effect of SPARC, we treated human OvCa cells SKOV3 and OVCAR3 with increasing concentrations of rSPARC and found that SPARC exhibited concentration-dependent inhibition of FA uptake in both cell lines (Fig. 6f). Moreover, treating SKOV3 and OVCAR3 with rSPARC, significantly decreased the expression of FA transporters FABP4 and CD36 as revealed by immunofluorescence staining and quantification of the fluorescence intensity (Fig. 6g, h). To determine whether the increased FA uptake in ID8 cells was associated with changes in FA metabolism, we found that the transcript levels of enzymes involved in β-oxidation of FA as carnitine palmitoyltransferase I (*Cpt1a, b*), acetyl-CoA acetyltransferase 2 (*Acat2*) a cholesterol acyltransferase, hydroxyacyl-CoA dehydrogenase (*Hadh)*, medium and short chain FA dehydrogenase, carnitine O-octanoyl transferase (*Crot*), acetyl-CoA acyltransferase 1 (*Acaa1*), ketoacyl-CoA thiolase 2 (*Acaa2*), and solute carrier family 25 (carnitine/acylcarnitine translocase member 20, *Slc25a20)* are increased in ID8 cells co-cultured with *SP*^*–/–*^ adipocytes compared with either ID8 cultured alone or with *SP*^*+/+*^ adipocytes (Fig. 6i).

### Effect of loss of stromal-SPARC on lipid metabolism in ID8 omental nodules

The enhanced growth and metabolic adaptation of OvCa that grow in adipose-rich peritoneal TME have been attributed to unconventional metabolism characterized by increased rates of the oxidation (β-oxidation) of FA released from adipocytes’ lipolysis [3]. FA and glycerol released from adipocytes are taken up by tumours cells, where FAs are used for β-oxidation, whereas glycerol may either be converted to glucose through gluconeogenesis or directly feeds into the glycolytic pathway providing energy for cellular metabolism [3, 39]. Therefore, we determined the effect of host-SPARC on the metabolic adaptation of ID8 omental tumour nodules developing in *SP*^*–/–*^ and *SP*^*+/+*^ mice. We found significant upregulation in the above mentioned enzymes involved in β-oxidation with exception of *Cpt1* in ID8 tumours growing in *SP*^*–/–*^ mice compared with those in the *SP*^*+/+*^ (Fig. 6j). Concomitantly, tumours growing in *SP*^*–/–*^ mice exhibited low levels of acylcarnitine conjugated lipids as palmitoyl carnitine and stearoyl carnitine suggesting reduced substrate availability due to enhanced lipid oxidation by the aforementioned enzymes. Consistently, significantly increased levels of 3-dehydrocarnitine, an intermediate of carnitine degradation were observed in in *SP*^*–/–*^ tumours consistent with a tumour metabolic phenotype. In addition, multiple monoacyl glycerols including 1-oleoylglycerol accumulated in *SP*^*–/–*^ tissues indicating a change in complex lipid hydrolysis evidenced by elevated levels of glycerol (Supplement Figure 9). These data suggest a novel significant role of adipocyte SPARC on metabolic reprogramming of OvCa cells in the unique peritoneal milieu.

### Inhibitory effect of SPARC on adipogenic differentiation

**Fig. 7:**
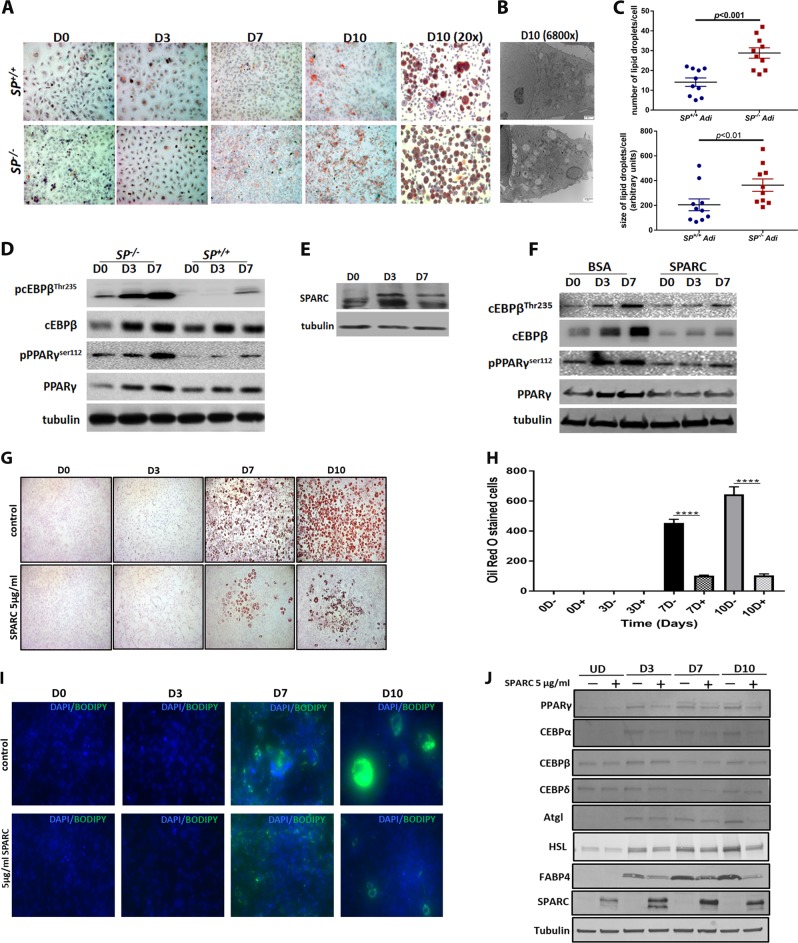
SPARC exerts anti-adipogenic effect on omental adipocytes and 3T3-L1 cells. **a** Oil Red O (ORO) staining of differentiating *SP*^*+/+*^ and *SP*^*–/–*^ pre-adipocytes (×10 magnification). **b** *SP*^*–/–*^ adipocytes exhibit increased size and number of fat droplets day 10 (D10) post-differentiation as shown by ORO (×20 magnification) and electron microscopy (×6800). **c** Plots indicate mean ± SEM of the number (upper) and size (lower) of lipid droplets quantified in EM images (10 cells/experimental condition). *P* values are determined by Student's t-test. **d** Western blots showing the kinetics of expression of adipogenic transcription factors during differentiation of *SP*^*+/+*^ and *SP*^*–/–*^ pre-adipocytes. **e** The expression of SPARC protein during differentiation of *SP*^*+/+*^ adipocytes. **f** Effect of SPARC (5 µg/ml in PBS–0.4% BSA) on the expression of the adipogenic transcription factors during differentiation of *SP*^*+/+*^ adipocytes. Tubulin was used as loading control. **g** Confluent 3T3-L1 pre-adipocytes (D0) were differentiated either in the presence or absence of 5 μg/ml rSPARC up to day 10. The cells were harvested at days 0, 3, 7, and 10. Intracellular lipids were stained with ORO. **h** Bars (right) represent means ± SEM of the quantification of ORO-stained cells solubilized with 100% isopropanol for 5min and measuring the absorbance A_492_ nm. *p < 0.0001, two-way ANOVA with Sidak post-hoc test. **h** Images of Bodipy fluorescent staining of intracellular lipids in differentiating 3T3-L1 pre-adipocytes in the presence or absence of 5 μg/ml rSPARC. **i** Western blots showing the expression of SPARC and the adipogenic differentiation markers in differentiating 3T3-L1 cells in the presence or absence of SPARC

The above described data prompted us to investigate the effect of SPARC on omental adipocytes’ differentiation and plasticity especially that the anti-adipogenic function of SPARC was earlier reported [25, 29, 40, 41]. Omental tissues isolated from age-matched *SP*^*–/–*^ and *SP*^*+/+*^ female mice did not exhibit discernible macroscopic or microscopic differences. Omental adipocytes were isolated from the buoyant layer after digestion and centrifugation of omental tissues (Supplement Figure 1A). *SP*^*–/–*^ adipocytes exhibited increased lipid droplets compared with *SP*^*+/+*^ (Supplement Figure 1A)^.^ Pelleted stromal cells were composed mainly of adipocytes, pre-adipocytes, and vascular smooth muscle cells [29] and exhibited fibrobalstoid phenotype in standard culture as stained with α-smooth muscle actin (Supplement Figure 1C). When confluent monolayers of *SP*^*+/+*^ and *SP*^*–/–*^ omental stromal cells were exposed to adipogenic growth medium (ADM) for 7 days D7, *SP*^*–/–*^ exhibited faster adipogenic differentiation, with increased accumulation of intracellular lipids as determined by Oil Red O (ORO, Fig. 7a), and electron microscopy with significantly larger numbers and size of lipid droplets (Fig. 7b, c). Fully differentiated *SP*^*–/–*^ adipocytes exhibited significantly higher levels of adipogenic TFs PPARγ, and c/EBPβ than *SP*^*+/+*^ adipocytes (Fig. 7d). SPARC protein exhibited differential expression in differentiating *SP*^*+/+*^ adipocytes being highest after 3 days (D3), in adipogenic medium and declined to basal levels in fully differentiated adipocytes at D7 (Fig. 7e). Exogenous SPARC suppressed the expression of adipogenic factors when added to differentiating *SP*^*–/–*^ adipocytes (Fig. 7f). Independently, treating 3T3-L1 pre-adipocytes with rSPARC (5 μg/ml) for 10 days significantly inhibited the accumulation of intracellular lipids as determined by ORO (Fig. 7g) and Bodipy fluorescent staining (Fig. 7h). Consistently, SPARC inhibited the expression of adipogenic TFs PPARγ, cEBPα, and cEBPβ, as well as markers of adipogentic differentiation HSL, ATGL, and FABP4 (Fig. 7i). Interestingly, consistent with our observation with *SP*^*+/+*^ adipocytes, SPARC protein expression increased in differentiating 3T3L adipocytes by D3 then decreased to basal levels by D7 and D10 (Fig. 7j). In addition, treatment with rSPARC decreased the mRNA levels of the master regulator of differentiation *Pparg* and *Cebp* isoforms, as well as markers of early and late differentiation as *Fasn, Adipoq, lep, Cd36, Acly, Scd1, Acaca, Pnpla2/Atgl1, Lipe/Hsl*, and *Screbp-1c* (Supplement Figure 10). These data further support the effect of SPARC inhibiting omental adipocyte differentiation and in accord with earlier reports of the anti-adipogenic effect of SPARC on different adipocyte and mesenchymal stem cell niches. Importantly, when these adipocytes are challenged by OvCa cells, SPARC suppresses their interaction with cancer cells and consequently inhibits the acquisition of CAA phenotype that fosters OvCa omental dissemination and colonization.

## Discussion

In the present study, we expand our knowledge on the biological roles of SPARC in OvCa by investigating its role in regulating the interactions between OvCa cells and omental adipocytes. Our findings in the present study indicate that SPARC exerts a tumour-suppressor effect on OvCa cells in part through inhibiting their interactions with omental adipocytes; the main site of metastasis of OvCa [3, 42, 43], and the most common cause of mortality in OvCa patients [43].

We used multipronged approach employing human and murine OvCa cell lines, as well as primary human and murine omental adipocytes in single and co-culture. We confirmed the inhibitory effect of SPARC by using primary adipocytes from *SP*^*–/–*^ and *SP*^*+/+*^ mice in single and co-cultures with murine ID8 cell line, a model system that is extensively used in syngeneic models of OvCa in normal and transgenic mice C57B6 background and is well characterized as has been reported in ~145 publications. However, ID8 cell line may not be considered a faithful representation of HGSC because it was originally derived by spontaneous transformation of high passage murine ovarian surface epithelium [44]. To circumvent this limitation of the mouse model, and to ascertain that our findings are not limited to the phenotype of one cell line or model system, we complemented our mechanistic and phenotypic studies with three human OvCa cell lines in mono- and co-cultures with human omental (pre)adipocytes, as well as human tumour specimens, as well as genetic manipulation of SPARC in human primary omental (pre)adipocytes and human OvCa cells. We further demonstrated the role of SPARC in omental adipocyte differentiation as *SP*^*–/–*^ adipocytes exhibited accelerated adipogenic differentiation and significantly accumulated more lipids than *SP*^*+/+*^ adipocytes. This finding is consistent with earlier report of increased tendency of *SP*^*–/–*^ bone marrow cells for adipogenic differentiation [25, 29, 40].

Indeed, the use of the 3D co-cultures of OvCa cells and adipocytes provided a useful tool for mechanistic and functional studies of tumour cells and adipocytes with monitoring concomitant changes in both cell types in response to genetic manipulations and pharmacologic inhibitors. We confirmed the specificity of the inhibitory effect of adipocyte-SPARC on the functional cross-talk between OvCa cells and adipocytes independent of contribution of other cells in the TME and the complex host background. This approach first confirmed the direct autocrine and paracrine effects of SPARC on tumour cell–adipocyte interactions. Adipocyte-SPARC exerted a suppressor effect at multiple levels. First, adipocyte-SPARC decreased homing, adhesion, proliferation of OvCa cells to omental adipocytes and inhibited their invasiveness. We showed that these effects were due to the effects of SPARC mitigating inflammatory secretome as evidenced by functional blocking antibodies. These are in accord with our earlier reports of the anti-inflammatory role of SPARC in OvCa [6–8]. Second, adipocyte-SPARC, as well as rSPARC suppressed the phenotypic switch of omental adipocytes and their acquisition of CAA inflammatory phenotype through suppression of OvCa cell-induced activation of cEBPβ, NFκB, and AP-1 TFs and their downstream inflammatory and metabolic effects. Pharmacologic inhibitors of these TFs not only phenocopied the effect of rSPARC on FFA release from adipocytes in mono-and co-cultures with OvCa cells, but they also mitigated the increase FFA release from *SP*^*–/–*^ adipocytes in co-cultures with ID8 cells. Inflammatory chemokines as TNFα, IL-2, and IL-6, were reported to increase lipolysis and negatively affect metabolic homeostasis [15, 45–48]. Moreover, the reciprocated transactivation between cEBPβ, NFkB, and AP-1 in myriad physiological and pathological contexts including lipid homeostasis, differentiation, inflammation, and cancer has been established and involves the transcription of multiple common factors including the enzymes, and chemokines studied herein. Our data using pharmacologic inhibitors of NFkB further supported the transactivation loop between NFkB and cEBPβ that has been earlier reported and confirmed [16, 21]. The association of these TFs with aggressiveness of many cancers, including OvCa, have been reported [28, 49–51]. Our data using patients’ specimens indicated the progressive increase and nuclear localization of these TFs as a function of OvCa progression. Importantly, their expression inversely correlated with the expression of SPARC protein in the cancerous compartment, as well as in the juxta-tumoral adipocytes in two independent patients’ cohorts. Survival data curated from The Cancer Genome Atlas (TCGA) revealed that the expression of cEBPβ, cJun, cFos, and NFkB1 were associated with poor patients’ survival (Supplement Figure 11). SPARC protein expression in the OvCa cells negatively correlates with nuclear localization of these TFs in patients’ specimens and in syngeneic tumours growing in *Sparc*-deficient mice. In tumour specimens with adjacent omental tissues, we show that the negative correlation between both tumour and adipocyte SPARC with the nuclear localization of these factors in tumour cells and juxta-tumoral adipocytes. SPARC transcript was not correlated with patients’ survival, most probably due to the distinctive compartmentalization of SPARC protein expression in patients’ tumours. In addition, our unpublished data indicated that genetically manipulating SPARC in SPARC-proficient CAOV3 cell line by overexpression or knockdown inversely correlated with malignant phenotype in vitro and peritoneal dissemination and mouse survival when they were injected ip in nude mice. Furthermore, we provide evidence that SPARC exerts a tumour-suppressor effect in OvCa linking inflammation to metabolic programming, a process termed “metaflammation” that has been implicated in diseases associated with inflammation and perturbed bioenergetics as diabetes, obesity and aging [52]. Together with our recent report that SPARC inhibits metabolic plasticity and mitochondrial bioenergetics in OvCa [53], our data underscore the multi-faceted suppressor role of SPARC in limiting peritoneal dissemination of OvCa.

Using OvCa cell-adipocytes co-cultures, we found that SPARC inhibited the increased lipolysis and FFA release from adipocytes concomitant with inhibition of increased FA uptake by OvCa cells. We show that the effect of SPARC is due to differential effects on adipocytes and OvCa cells; not only inhibiting inflammation-induced lipolysis and FFA release in adipocytes, but also downregulating the surface expression of FA transporters CD36 and FABP4, both have been associated with poor prognosis in patients with OvCa [54–56]. FABP4 has been recently reported as hypoxia-regulated gene [56], and its expression in tumour endothelial cells is associated with increased angiogenesis, especially in low-grade stroma-rich tumours [57]. In addition, FABP4 has been reported as predictor of residual disease in HGSC [55] and its overexpression in patients’ tumours is associated with increased metastatic burden and poor survival [56].

As a consequence of increase FFA uptake, OvCa cells have been shown to rely on FA oxidation for energy production [3]. Recent reports [58] demonstrated the link between FA oxidation and cancer cell proliferation and survival through activation of salt-inducible kinase-2 (SIK2) in tumour cells co-cultured with omental adipocytes. Activated SIK2 phosphorylated acetyl-CoA carboxylase (ACC) and phosphatidyl inositol 3 kinase (PI3K) and, thus, simultaneously regulates both fatty acid oxidation and cancer cell proliferation and survival. Consistently, our recent report [53] showed that SPARC inhibits mitochondrial bioenergetics and ATP, glycolysis and ATP production, “metabolic plasticity” through inhibition of activation of adenosine monophosphate kinase, ACC, as well as mammalian target of rapamycin and its downstream targets; thus linking metabolic programming and mitochondrial dysfunction to cancer cell proliferation and survival. Together with our results herein, it is plausible that the effect of SPARC on cancer cell-adipocyte metabolic programming through an effect on SIK2, or through an effect on enzymatic processes that occur in the mitochondria including FA oxidation, leading to significant reactive oxygen species (ROS) generation and activation of the transcriptional inflammatory oncogenic machinery orchestrated by NFkB, cJun, and cEBPβ.

In summary, this is the first investigation of the role of adipocyte-SPARC in regulation of omental adipocytes and its impact on OvCa progression and omental metastasis. Our study provides novel information on the tumour-suppressor role of the SPARC in the regulation of OvCa-omental cross-talk and highlights the role of overexpression, as well as exogenous recombinant SPARC not only on mitigating the effect of loss of SPARC in adipocytes but on suppressing pro-tumorigenic and metabolic programming, thus making the omental adipocyte niche unfavorable for seeding and colonization of tumour cell. We show the effect of SPARC on activation of the main TFs orchestrating inflammation, adipocyte dysfunction, and cancer progression. Our data highlight the importance of SPARC protein as a promising therapeutic target in OvCa.

## Materials and methods

### Cell culture, plasmid transfections, and viral transductions

Murine (ID8) were earlier described [5, 7]. Human OvCa (SKOV3, OVCAR3, CAOV3) cell lines were originally from ATCC and were maintained at low passage and in complete growth media as earlier described [5, 7]. Cells were tested for mycoplasma once/month at Wake Forest Baptist Cell and Viral Vector Core Lab (CVVL). Primary human omental pre-adipocytes were obtained from Zen Bio, Inc. (Raleigh, NC, USA) and were maintained in omental pre-adipocytes medium and differentiated using adipocyte differentiation medium. Transient and stable overexpression of SPARC was done using adenoviral and retroviral vectors as previously described [7, 36]. Transduction efficiency was optimized to overcome negative selection due to the anti-proliferative effect of SPARC. SPARC depletion was done by transduction using lentiviral vectors with short-hairpin RNA targeting SPARC (shSPARC) with control shRNA targeting non-human target [36]. All were purchased from Sigma, TRC (TRCN000008710, TRCN000008709, and TRCN000008711). Culture media, supplements, antibiotics, and growth factor-reduced matrigel were from Invitrogen (Grand Island, NY) and BD Biosciences (Franklin Lakes, NJ). Recombinant human and murine SPARC were purchased from Peprotech (Rocky Hill, NJ, catalog# 120-36) and R&D Systems Inc. (Minneapolis, MN, catalog# 942-SP). Stock solutions of rSPARC from the same lot numbers were reconstituted in Dulbecco's phosphate buffered saline (DPBS)–0.1% bovine serum albumin (BSA) (1 mg/ml), aliquoted and stored at –08 ^o^C till used. The purity of SPARC was validated by SDS-PAGE under reducing and non-reducing conditions, and Commassie blue staining showing the abundance of the protein at the appropriate molecular weight along with the BSA (Supplement Figure 1). Unless otherwise stated, all reagents were purchased from Sigma Aldrich (St. Louis, MO) and ThermoFisher (Pittsburgh, PA).

### In vivo syngeneic model

*SP*^*+/+*^ and *SP*^*–/–*^ mice are maintained on C57BL/6 background for at least 10 backcrosses. Mice were housed in a specific pathogen-free (SPF) facility. All animal experiments were approved by IACUC of the University of Virginia (IACUC# 3879) and Wake Forest University Schools of Medicine (IACUC# A16-165). ID8 (4×10^6^/100 µl sterile PBS) ip injections in *SP*^*+/+*^ and *SP*^*–/–*^ mice was previously described [5, 7]. Dissected tumour tissues were either snap frozen in liquid nitrogen then stored at –80^o^C till used or fixed in 10% neutral zinc formalin and embedded in paraffin. For in vivo homing experiments, ID8-GFP cells (4×10^6^) were injected ip in 5-week-old female *SP*^*+/+*^ and *SP*^*–/–*^ mice [5]. Mice were euthanized at the time points indicated in figure legends, omenta were dissected, placed in wells of six-well plates, and ID8-GFP cell adhesion was determined using a fluorescent inverted microscope as earlier described [36]. GFP fluorescence intensity was quantified in five images/mouse omentum using Image J software. Background fluorescence was normalized to the fluorescence of images of omenta isolated from sham (PBS)-injected mice. For functional blocking experiments, mouse IL-6 (MAB406; 1 µg/ml), mouse TNFα (MAB4101, 1 µg/ml), CCL2/MCP-1 (AB-479-NA; 30 µg/ml), mouse anti-leptin (AF 498, 0.3 µg/ml), mouse CCL3/MIP1 (AF450, 0.3 µg/ml), mouse IL-2 (MAB702, 1 µg/ml), and VEGF (AF-493-NA; 1 µg/ml), were injected ip in *SP*^*+/+*^ and *SP*^*–/–*^ mice 30 min before ID8 injection. Normal isotype controls included rat anti-mouse IgG1 (MAB005), goat anti-mouse IgG (AB-108-C) injected in the same dose. All neutralizing antibodies were from R&D Systems. In some experiments, mice, athymic nude mice (Jackson Labs, Bar Harbor, ME) were injected subcutaneously (SC) with murine ID8 cells mixed with *SP*^*+/+*^ or *SP*^*–/–*^ adipocytes in a ratio of 1:2 (ID8:adipocytes). SC tumours were measured twice weekly by caliper for 6 weeks [5, 7, 36].

### Isolation of primary murine omental adipocytes

Omenta were isolated from euthanized 6- to 8-week-old female mice and were digested by collagenase/dispase for 2 h at 37 ^o^C with gentle agitation [25]. Slurries were allowed to separate into floating mature adipocytes (top layer) (Supplement Figure 1) and pellets mainly consisting of fibroblasts, smooth muscle cells, and pre-adipocytes [25, 41]. Mature adipocytes were immediately used for the assays involving murine cells. Adipocytes were maintained in adipocytes maintenance medium (ADMM, Zen Bio, Raleigh, NC) supplemented with 10% fetal bovine serum (FBS), antibiotic–antimycotic solution. Omental stromal cells were maintained in Dulbecco’s modified Eagle’s medium/F12 with 10% FBS and antibiotic–antimycotic solution. Adipogenic differentiation was initiated in confluent fibroblastoid omental stromal cells, by adipogenic medium (ADM, Zen Bio) for 3 days, after which they were switched to ADMM for further 10–14 days. The differentiation of adipocytes was compared by microscopic examination and stained with ORO staining and Bodipy fluorescent staining (Molecular Probes, ThermoFisher) [3].

### 3D omental cultures and in vitro proliferation, adhesion, and invasion assays

The 3D omental culture was assembled by plating 5 × 10^5^ fresh omental adipocytes in 500 µl of reduced growth factor–matrigel mixed with growth media (1:3) into a 24-well culture plate. For proliferation assays, ID8-GFP cells (5000 cells/100 µl serum-free media (SFM)–2% FBS) were added on top of adipocytes embedded in matrigel. Green fluorescence at A_488_ was determined daily after plating for 72 h. As controls, parallel experiments were performed using ID8-GFP cells grown under similar conditions in absence of adipocytes and/or matrigel. The number of adipocytes was determined by parallel experiments counting the number of adipocytes after sorting out GFP-labeled cancer cells before, during, and at the end of corresponding experiments. For proliferation experiments (~72 h), parallel experiments were performed as follows: for *SP*^*–/–*^ and *SP*^*+/+*^ adipocytes and ID8-GFP cells, another parallel experiment in which adipocytes in matrigel were separated from ID8-GFP cells by 0.4 µm trans-well inserts (ID8 cells added in the top chambers and the inserts were touching adipocytes). ID8 proliferation was determined by measuring A_488_ fluorescence and cell counting by Trypan blue exclusion. Adipocytes in the bottom chambers were trypsinized and counted. Similarly, human omental pre-adipocytes embedded in matrigel were allowed to differentiate and OvCa-GFP expressing cells were added on the top chamber of trans-well inserts (0.4 µm) directly placed on the adipocytes plugs, only separated by the membranes. At the time points indicated in the figures and figure legends, OvCa cells were collected and their proliferation were determined by cell counting and Trypan blue exclusion, as well as measuring the GFP fluorescence. Adipocytes in the bottom were trypsinized and counted by Trypan blue exclusion. We did not find changes in the adipocytes numbers between the experimental conditions up to 72 h.

For in vitro homing experiments, ID8-GFP cells (1 × 10^5^) were added in the top chamber of 8 µm-pore trans-well inserts and were allowed to migrate towards *SP*^*–/–*^ and *SP*^*+/+*^ primary adipocytes or *SP*^*–/–*^ and *SP*^*+/+*^ adipocytes in the bottom chambers for 6 h at 37 °C. For invasion assays, 1×10^5^ OvCa cells/100 µl SFM were added on top of matrigel-coated 8 µm-pore trans-well inserts, and incubated at 37 ^o^C for 6 h with SPARC-deficient and SPARC-proficient omental adipocytes or their CM in the bottom chambers of the trans-wells [3, 59, 60].

### Antibodies, reagents, and western blots

Monoclonal and polyclonal antibodies against total and phosphorylated cEBPβ, PPAR-γ, HSL, p-HSL (ser660), cJun, p65NFκB, ATGL, human and murine SPARC, α-smooth muscle actin (α-sma), and β-tubulin antibodies were obtained from Cell Signaling Technology (Danvers, MA, USA), Abcam (Cambridge, MA), and Santa Cruz (Santa Cruz, CA). Cells were lysed and protein concentration were determined as earlier described [7, 36]. Cellular proteins (20 µg) were resolved by 4–20% SDS-PAGE, transferred to polyvinylidene difluoride (PVDF) membranes (Bio-Rad, Hercules, CA, USA), and probed with primary and the appropriate fluorescent- and horseradish peroxidase (HRP)-conjugated secondary antibodies (Licor, NE and Abcam). Blots were visualized using Odyssey 3v and Amersham Imagers.

### Luciferase reporter assays

NFκB and AP-1 luciferase reporters were described previously [35, 36]. The cEBPβ responsive luciferase vector (pGL2–5xcEBPβ-TK-Luc) and matching empty vector control [21] were kindly provided by Dr. Xianjun Fang at Medical College of Virginia, Richmond, VA. Differential luciferase reporter assays in co-cultures of OvCa cells and adipocytes were performed as earlier described [35, 36]. For investigation of the TF reporter activity, ID8 and omental adipocytes were transfected with the luciferase reporter plasmids using Fugene 6 (Promega, Madison WI) 24 h prior to co-culture. As an internal control for transfection efficiency, cells were co-transfected with TK-*Reinella*-luciferase plasmid (at a ratio of 20:1 firefly:TK-*Reinella*). Cells were co-cultured for 24 h and the luciferase activity was measured using Dual Luciferase Reporter Assay kit (Promega) as per manufacturer’s instruction. Results were further normalized to DNA content of the cells determined by CyQuant assay to correct for changes in cell proliferation/experimental condition [35, 36, 59].

In some experiments, the following inhibitors were included in the co-cultures of OvCa cells and adipocytes: NFkB inhibitory peptides for RelA/NFkB p65 (p Ser276, NBP2-26505, 50 µM), which functions as a p65 decoy inhibiting Ser276 phosphorylation of RelA, and NFkB p50 (NLS, NBP2-29323, 50 µM) inhibitory peptide that blocks p50 nuclear translocation, as well as their control peptide (NBP2-29334, 50 µM). NFkB inhibitory peptides were purchased from R&D Systems. JNK inhibitor SP600125 was from SelleckChem, Inc. (Houston, TX) supplied as10 µM in dimethylsulfoxide (DMSO). Of note is that most of the commercially available cEBPβ inhibitors function by virtue of their anti-inflammatory effect [61] and were also reported to inhibit p65RelA subunit of NFkB [62]. Thus, we used NFkB inhibitors and confirmed their inhibitory effects on cEBPβ activity.

### Cytokine, chemokine, and metabolic assays

The levels of cytokines/chemokines in the CM of different experimental conditions were determined using the appropriate species-specific commercial kits from R&D Systems, and RayBiotech Inc., as per the manufacturer’s recommendations. Intracellular fatty acids were determined by Bodipy fluorescent staining (Molecular Probes) and oil red staining. Fatty Acid Fluorometric Assay and uptake assay kits were from Cayman Chemicals (Ann Arbor, MI) and Abcam.

### Human ovarian tumour tissues

Human OvCa TMA was obtained from the University of Virginia Cooperative Human Tumor Network (CHTN). We included results from serous papillary and poorly differentiated subtypes form CHTN TMA. Thirty-five de-identified human OvCa tumour tissues with the adjacent omental tissues were obtained from Wake Forest Tumor Tissue and Pathology Shared Resources (WF-TTPSR, IRB#IRB00036014). The clinical data of the samples are summarized in Supplement Table 1.

### Immunohistochemistry

Monoclonal and polyclonal antibodies against SPARC, p65NFκB, cEBPα, cJun, CD36 and FABP4 and horse raddish peroxidase (HRP)- and fluorescent-labeled secondary antibodies were purchased from sources described in Supplement Table 2. For immunohistochemistry paraffin, HRP secondary antibodies were used and signal was developed with ImmPACT DAB Peroxidase (HRP) Substrate (Vector Labs, Burlingame, CA) and counterstained with hematoxylin. For immunofluorescence staining, appropriate Alexa-fluor 488 and 594 secondary antibodies were used and nuclei were counterstained with prolong anti-fade mounting media (Invitrogen). Nuclear and cytoplasmic expression of the aforementioned proteins in cancer cells and adipocytes were determined as earlier described [35, 36, 60]. Images were acquired using Aperio Scanscope (Leica Microsystems, Buffalo Grove, IL), and Olympus VS-110 Virtual Imaging System (Life Science Solutions, Center Valley, Pennsylvania). Digital image analysis of protein intensity and frequency was performed using Halo software (Indica Lab, Corrales, NM) and VisioPharm (Broomfield, CO) to segment tumour vs stroma and nuclear vs cytoplasmic and tissue alignment of sections stained with hematoxylin and eosin (H&E) with those stained colorimetric and fluorescent stains. We specifically focused on the areas that include tumour–adipocyte interface and classified the regions containing mainly adipocytes as “stroma” as guided by aligned matching H&E slides. Staining frequency and intensity were assigned arbitrary scores as earlier described [35, 36, 60]. Composite expression score (H-score) was calculated by multiplying the frequency and intensity scores [35, 36, 60]. For CD36 and FABP4 immunofluorescence staining, cells were seeded in duplicate wells LabTek eight-well slide chambers and were treated with SPARC (5 µg/ml) for 24 h. Immunostaining was carried out using rabbit anti-CD36 and FABP4 after fixing cells with 4% paraformaldehyde and permeabilization with Triton X-100. Alexa-fluor 594 secondary antibodies (Invitrogen) were used. Image acquisition was done using Leica AF6000 Modular System confocal microscope (Leica). Image acquisition, deconvolution, and maximum projection analysis were performed with the program LAS AF (Leica). Morphometric analysis was performed by Image J [53] in 10 random fields/experimental condition. Each experiment is performed three times.

### RNA isolation and qRT-PCR

Total RNA was extracted from cultured cells by RNeasy kits (Qiagen, Valencia, CA) and qRT-PCR was carried out using iScript cDNA Synthesis Kit and iQSYbr Green Supermix and Bio-Rad CFX thermal cycler (Bio-Rad, Hercules, CA). The primer sequences for mouse and human genes are summarized in Supplement Tables 3-4. Each experiment was performed in triplicate and repeated three times.

### Metabolomic profiling

Snap-frozen dissected omental tumour nodules that developed in *SP*^*+/+*^ and *SP*^*–/–*^ mice (*n* = 6/cohort) were prepared as previously described [63]. Briefly, samples were de-proteinized and protein-associated metabolites were extracted. The resulting extracts were analyzed by ultra-performance liquid chromatography and mass spectroscopy (UPLC-MS/MS) with positive and negative ion mode electrospray ionization, as well as gas chromatography and mass spectroscopy (GC-MS). Samples were analyzed on a Thermo-Finnigan Trace DSQ fast-scanning single-quadrupole mass spectrometer using electron impact ionization (EI) and operated at unit mass resolving power. Raw data were extracted, peak identified, QC processed, and normalized using proprietary Metabolon’s hardware and software. Compounds were identified by comparison with library entries of purified standards or recurrent unknown entities [63].

### Transmission electron microscopy (TEM)

Tumour tissue sections were processed for TEM at Wake Forest Baptist Medical Center (WFBMC) Imaging Core Facility according to standard protocols [64]. Sections were viewed with an FEI Tecnai Spirit TEM operating at 80 kV and images were acquired with an AMT 2Vu CCD camera. Image analysis was performed by counting the number of lipid droplets/cells in 10 cells/experimental condition. Lipid droplet size was measured in images ×1800 magnification by ImageJ. The averages of the area of lipid droplets/cell were calculated in 10 cells/experimental condition and was presented as arbitrary units.

### Statistical analysis

All other data were analyzed by two-tailed unpaired Student's *t*-test and one- and two-way analysis of variance (ANOVA) with Sidak or Tukey post-hoc tests. The association of the expression of different proteins were evaluated using the nonparametric Wilcoxon–Mann–Whitney and Kruskal–Wallis tests. Correlation between the expression scores in stained tissues were performed using Spearman’s correlation. Differences were deemed significant at *p* < 0.05. GraphPad Prism 7.0 (San Diego, CA).

## Supplementary information

Supplemental Material
