# Supplementary material for: Regulation of the bi-directional cross-talk between ovarian cancer cells and adipocytes by SPARC
Source: Oncogene. 2019 Feb 14;38(22):4366–83. doi: 10.1038/s41388-019-0728-3 (PMC6542715; doi:10.1038/s41388-019-0728-3)
Supplement: Supplementary file 1 — Supplemental Material [file 41388_2019_728_MOESM1_ESM.pdf]

# Supplement Figure 1

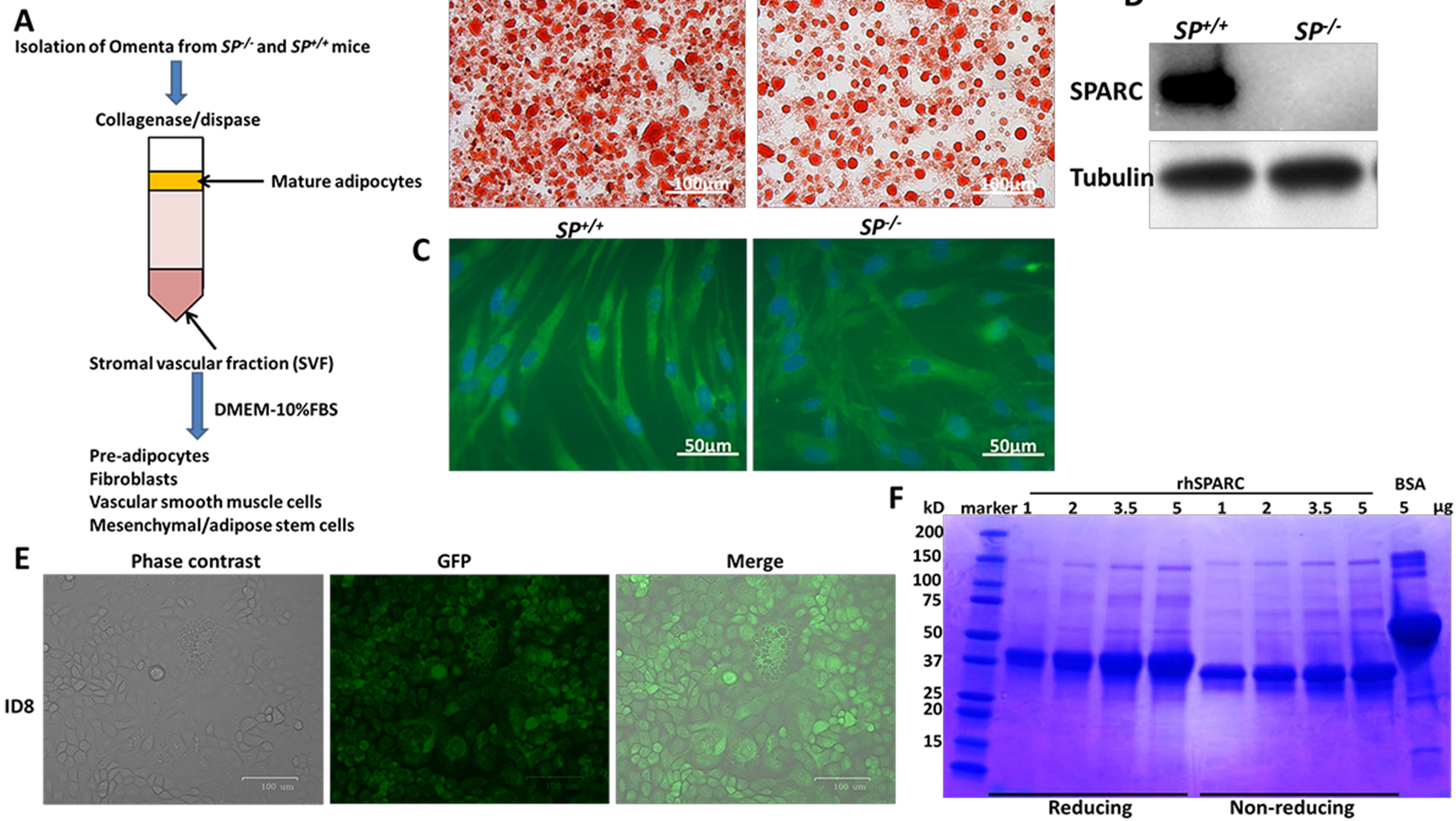

# Supplement Figure 2

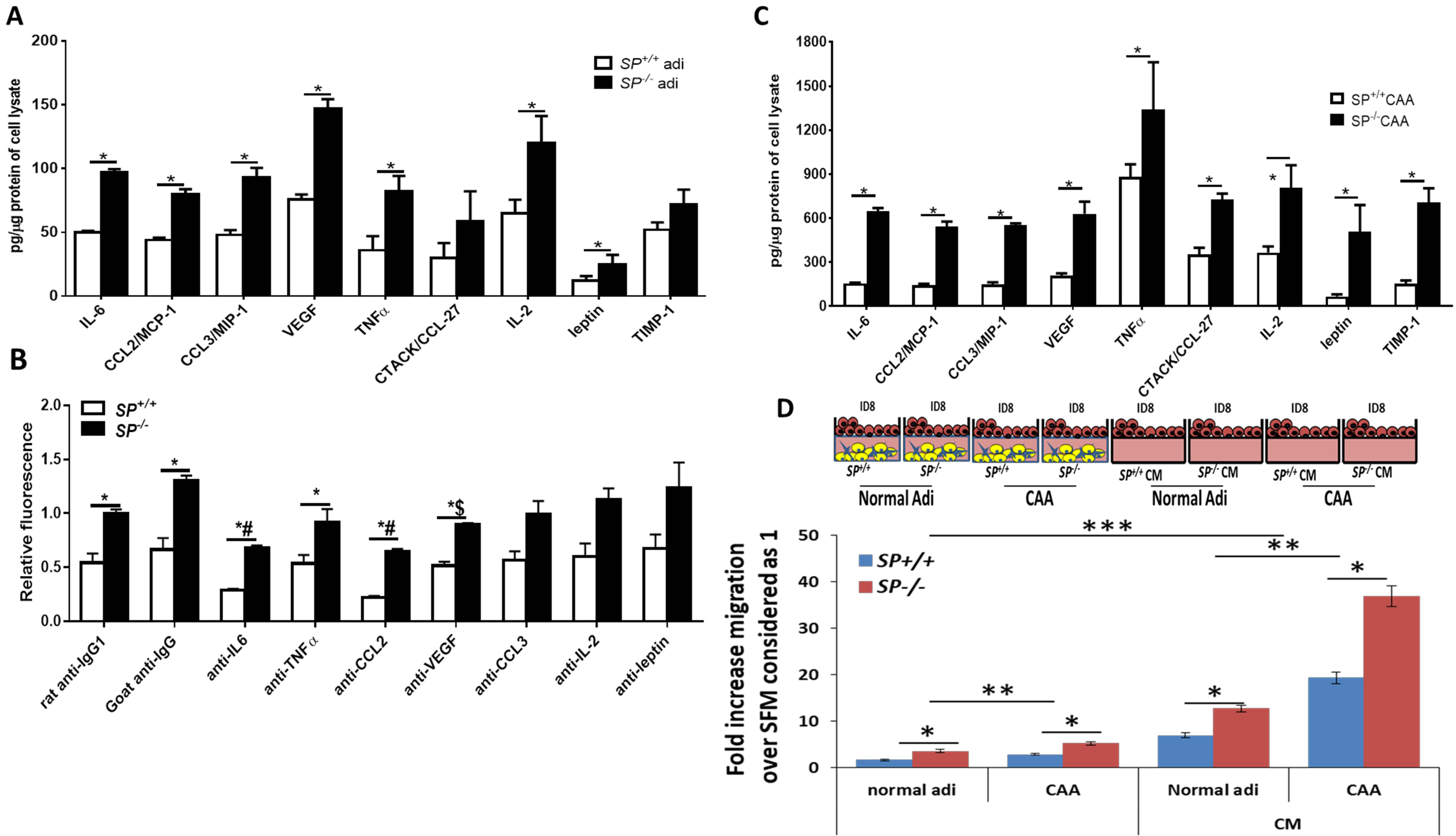

## Supplement Figure 3

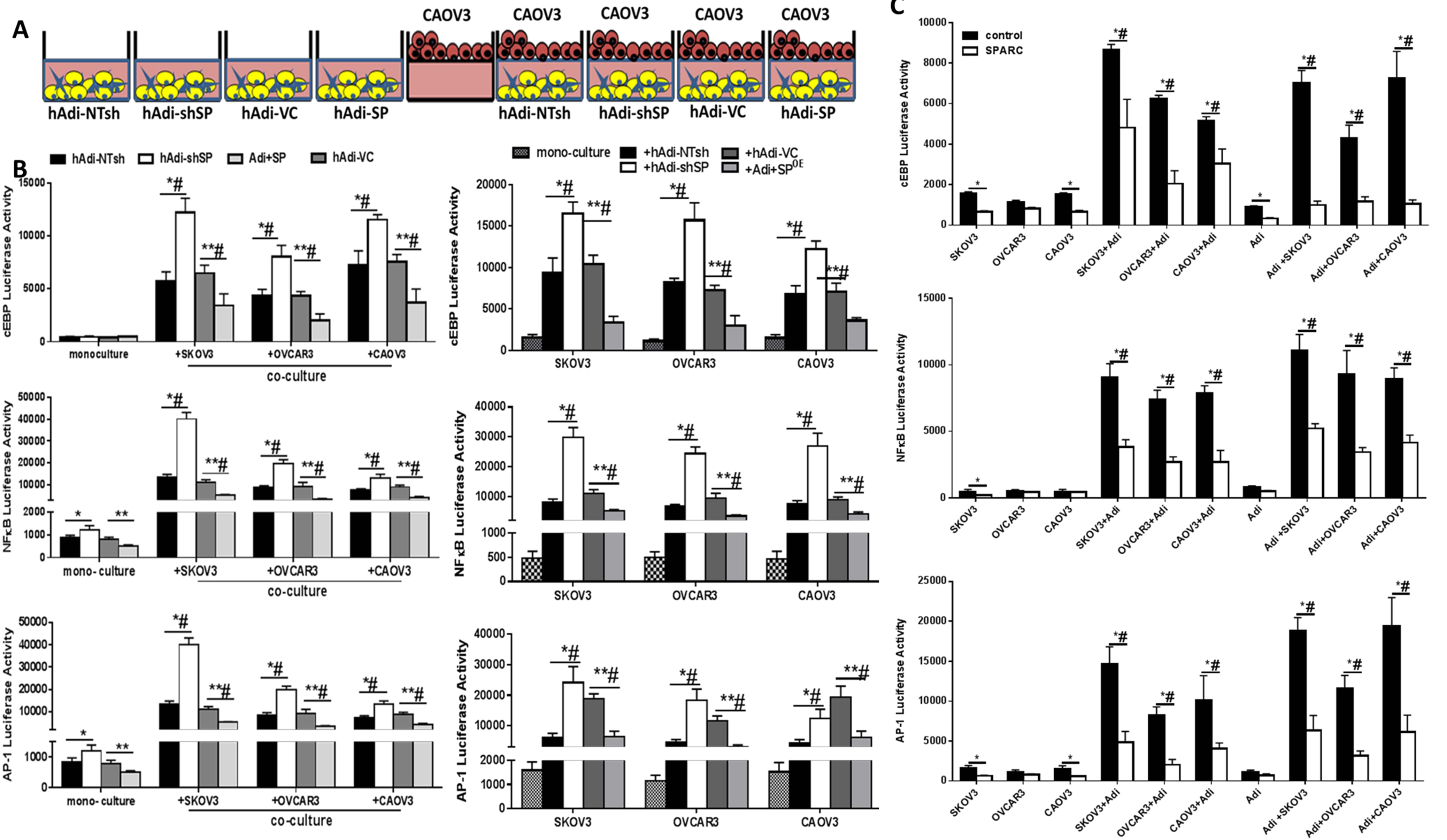

# Supplement Figure 4

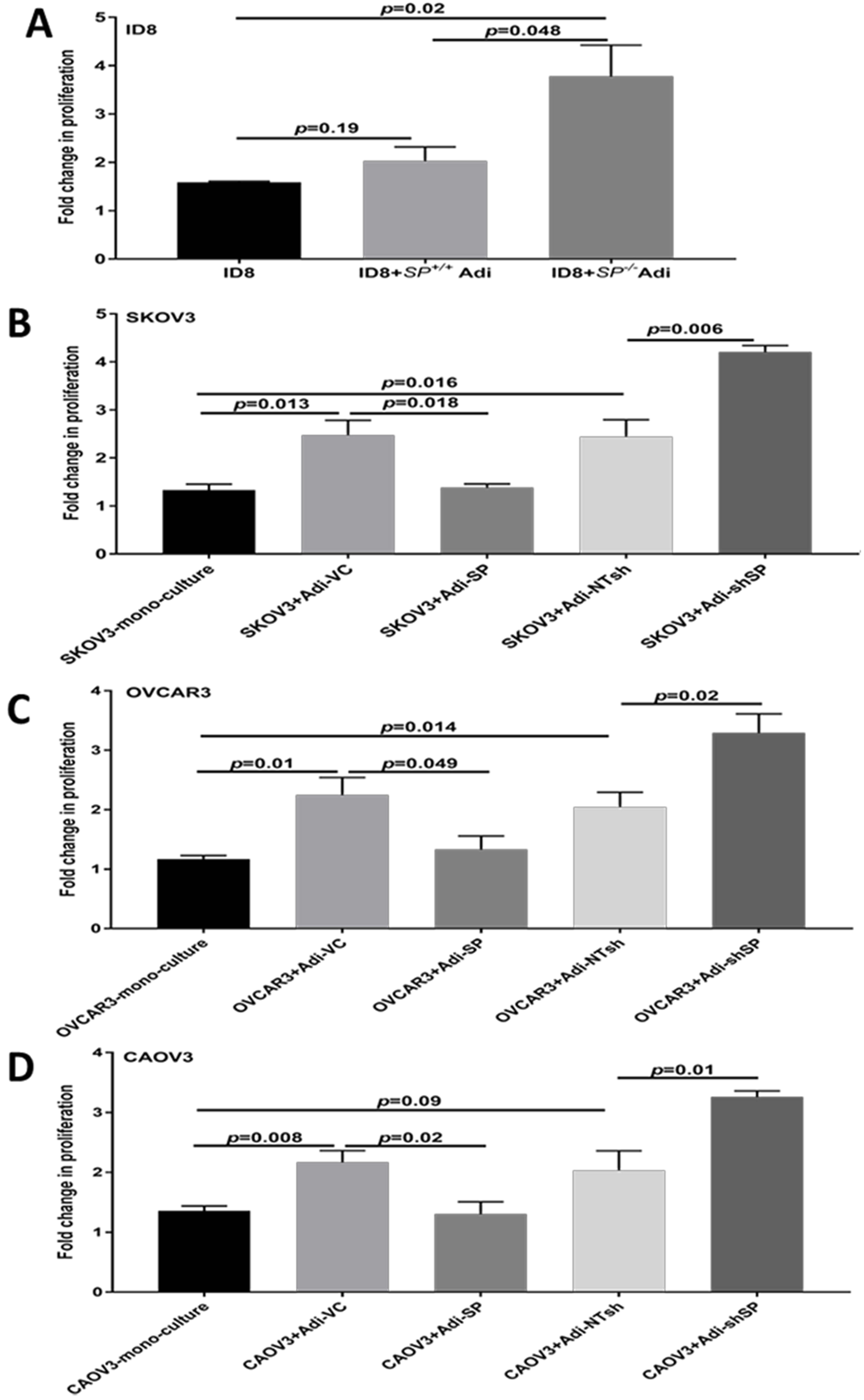

## Supplement Figure 5

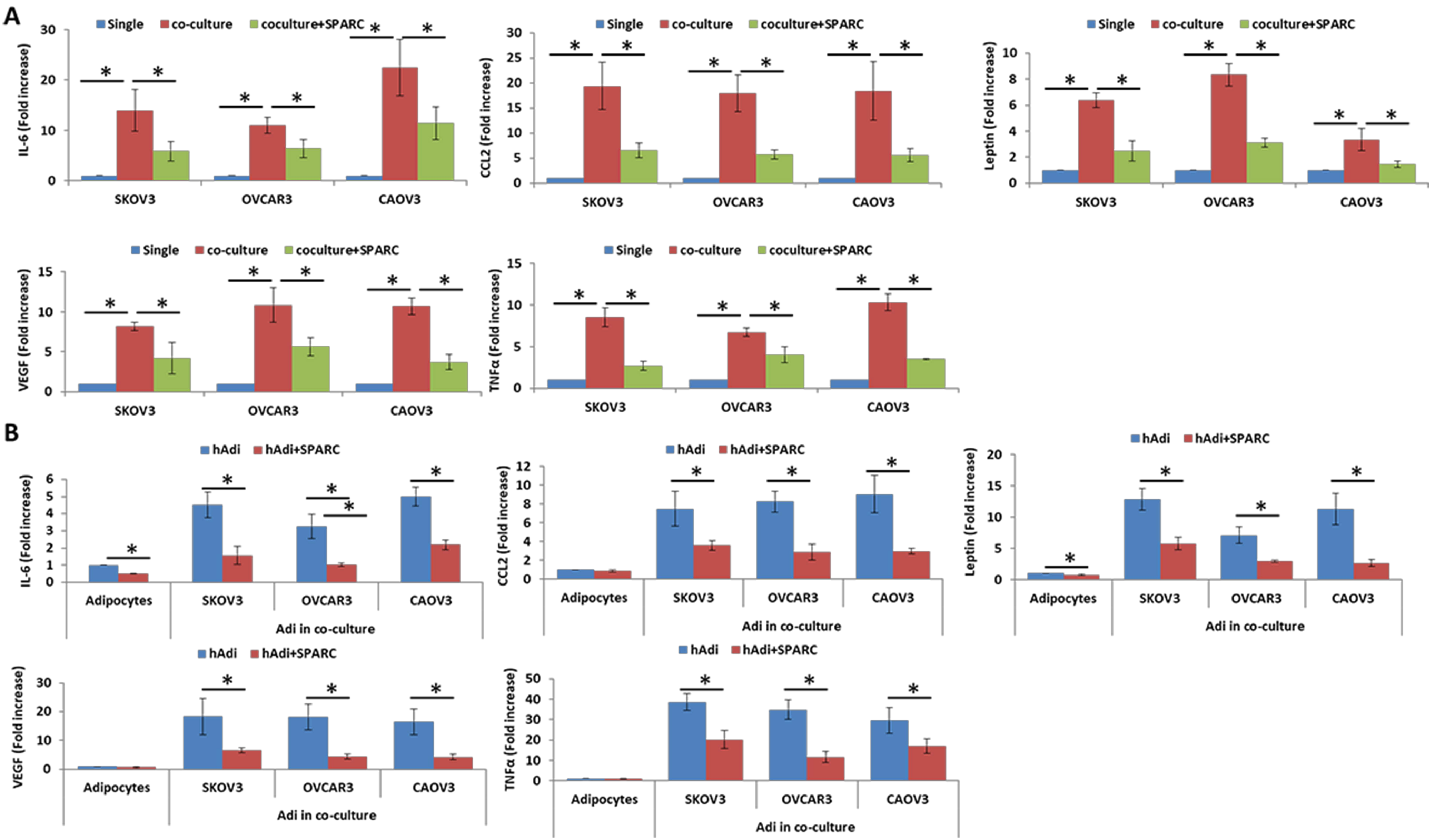

# Supplement Figure 6

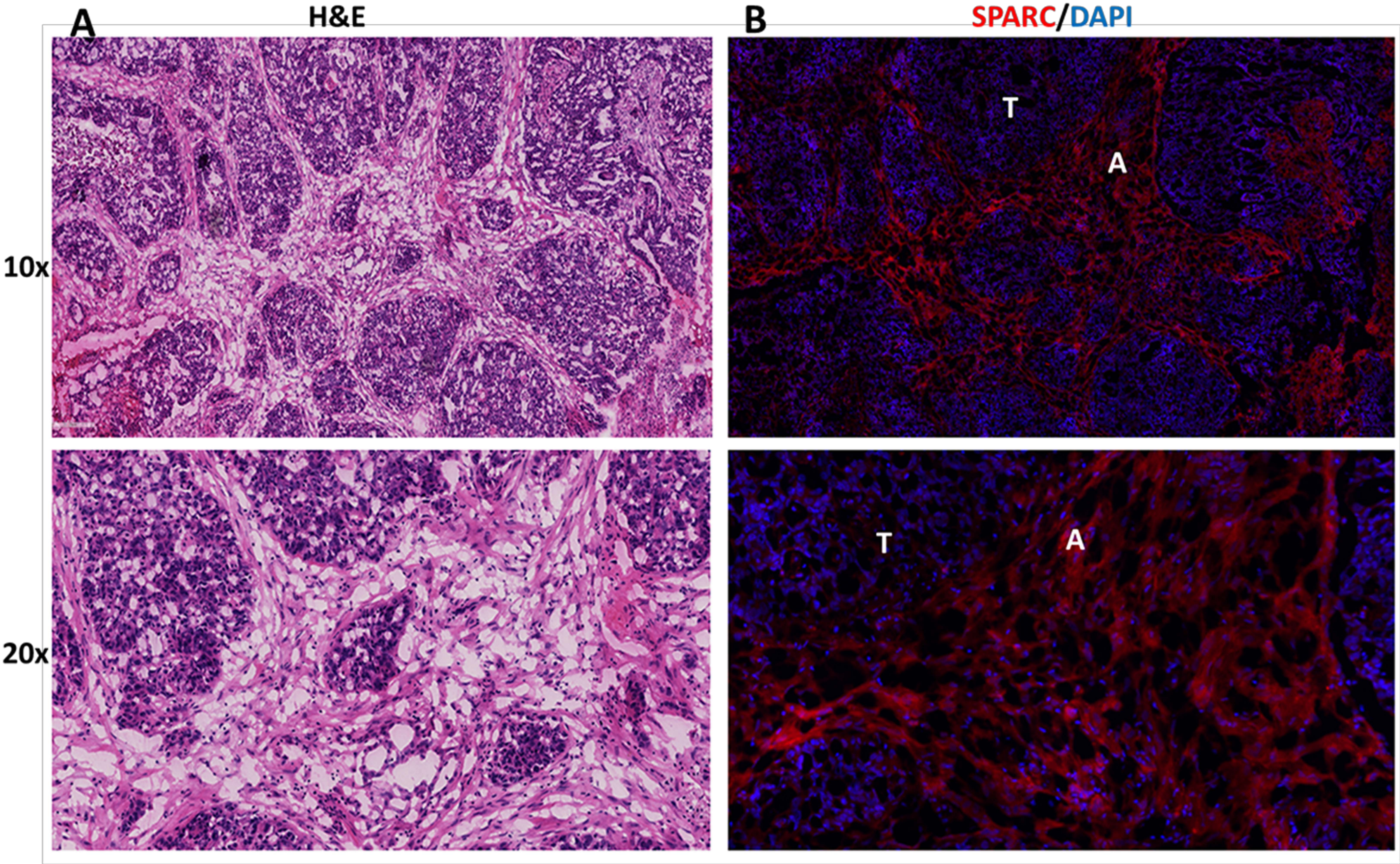

# Supplement Figure 7

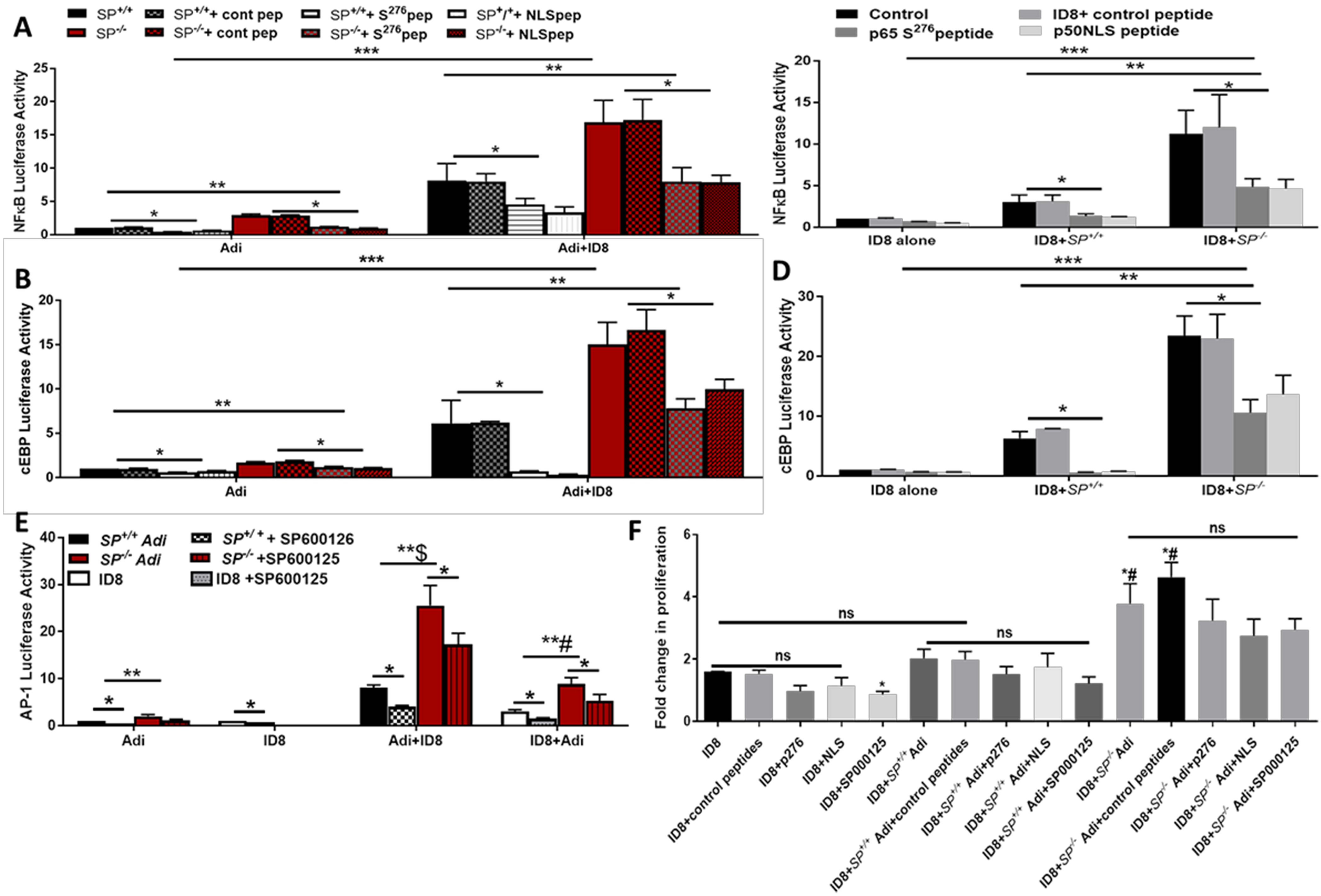

**Supplement Figure 8:**

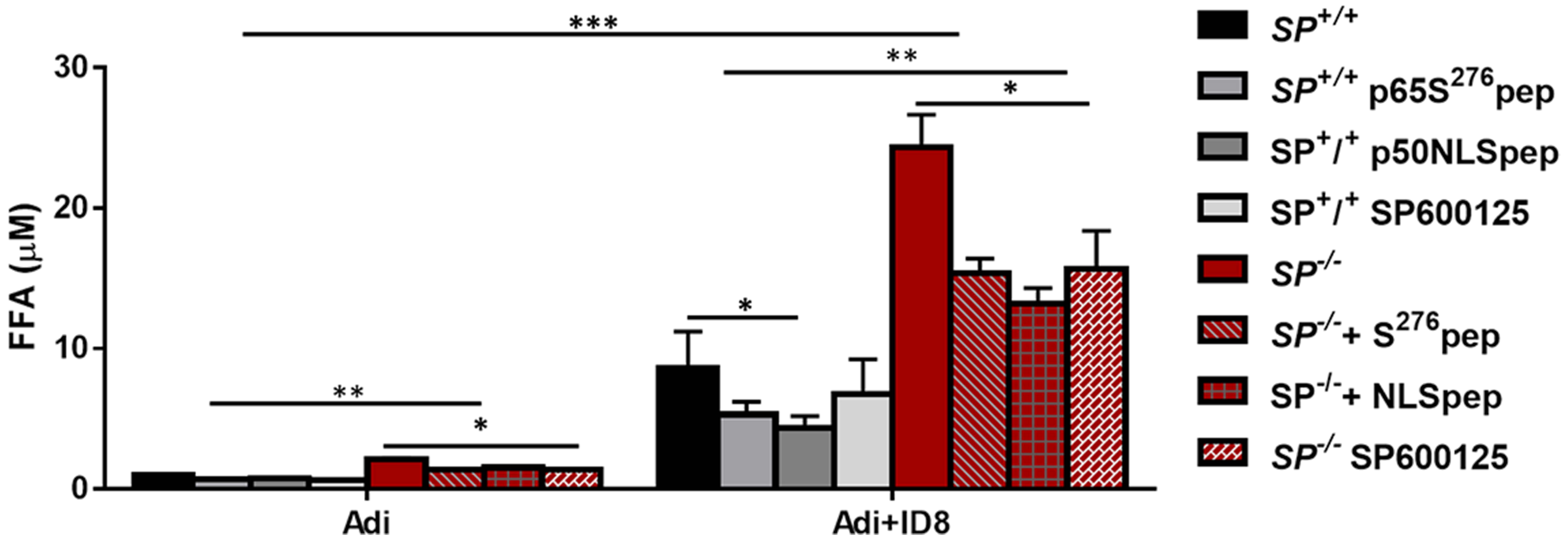

# Supplement Figure 9:

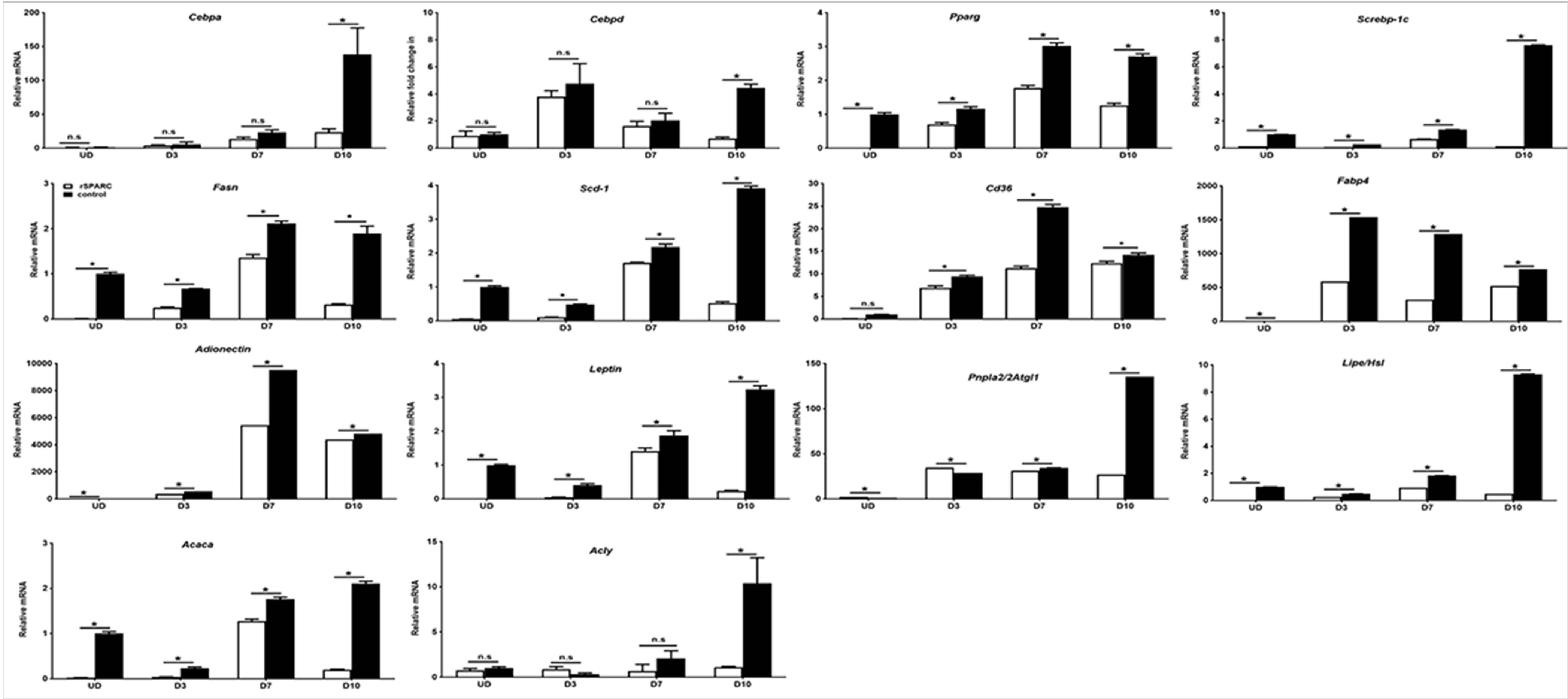

## Supplement Figure 10:

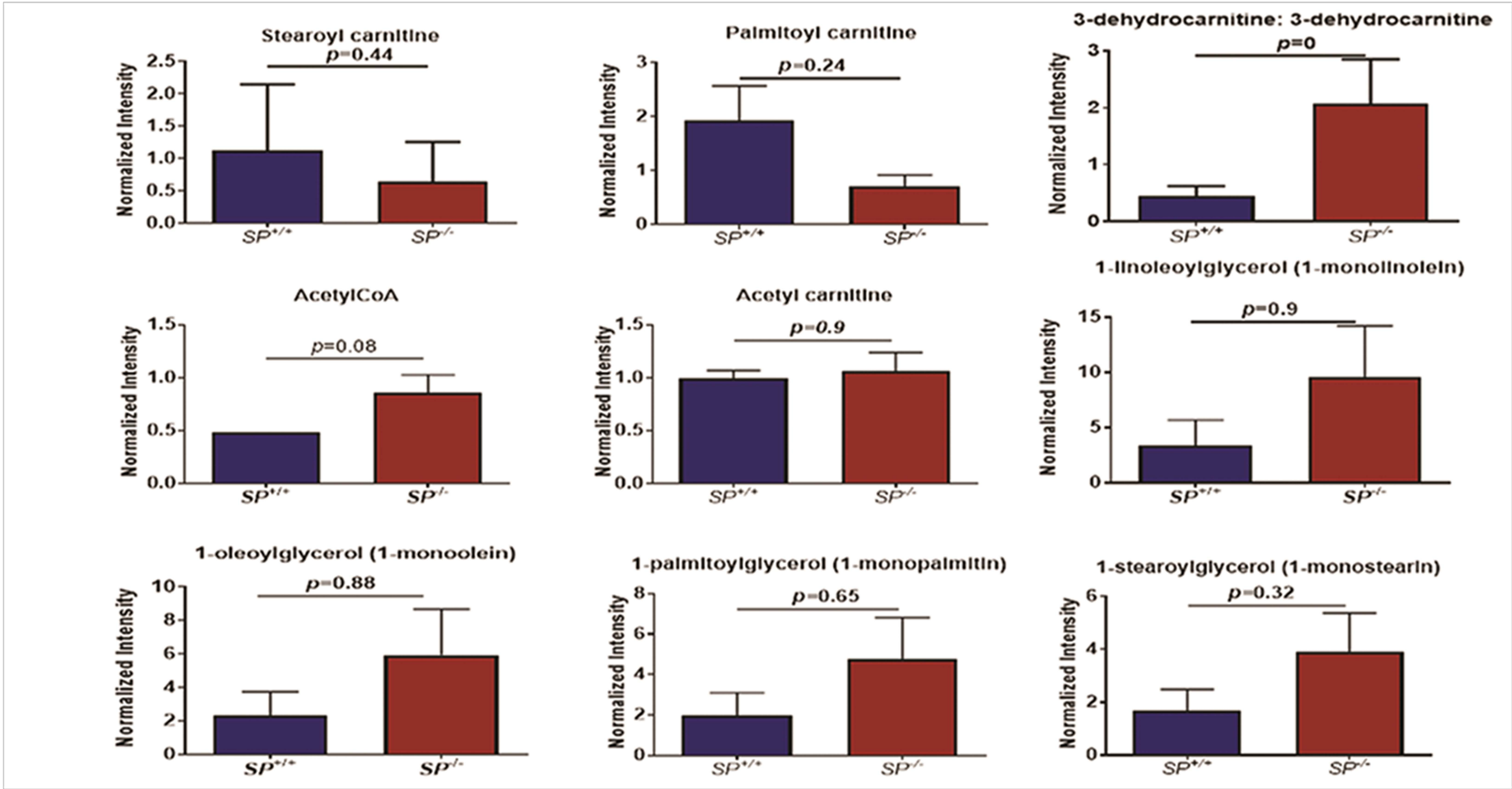

# Supplement Figure 11:

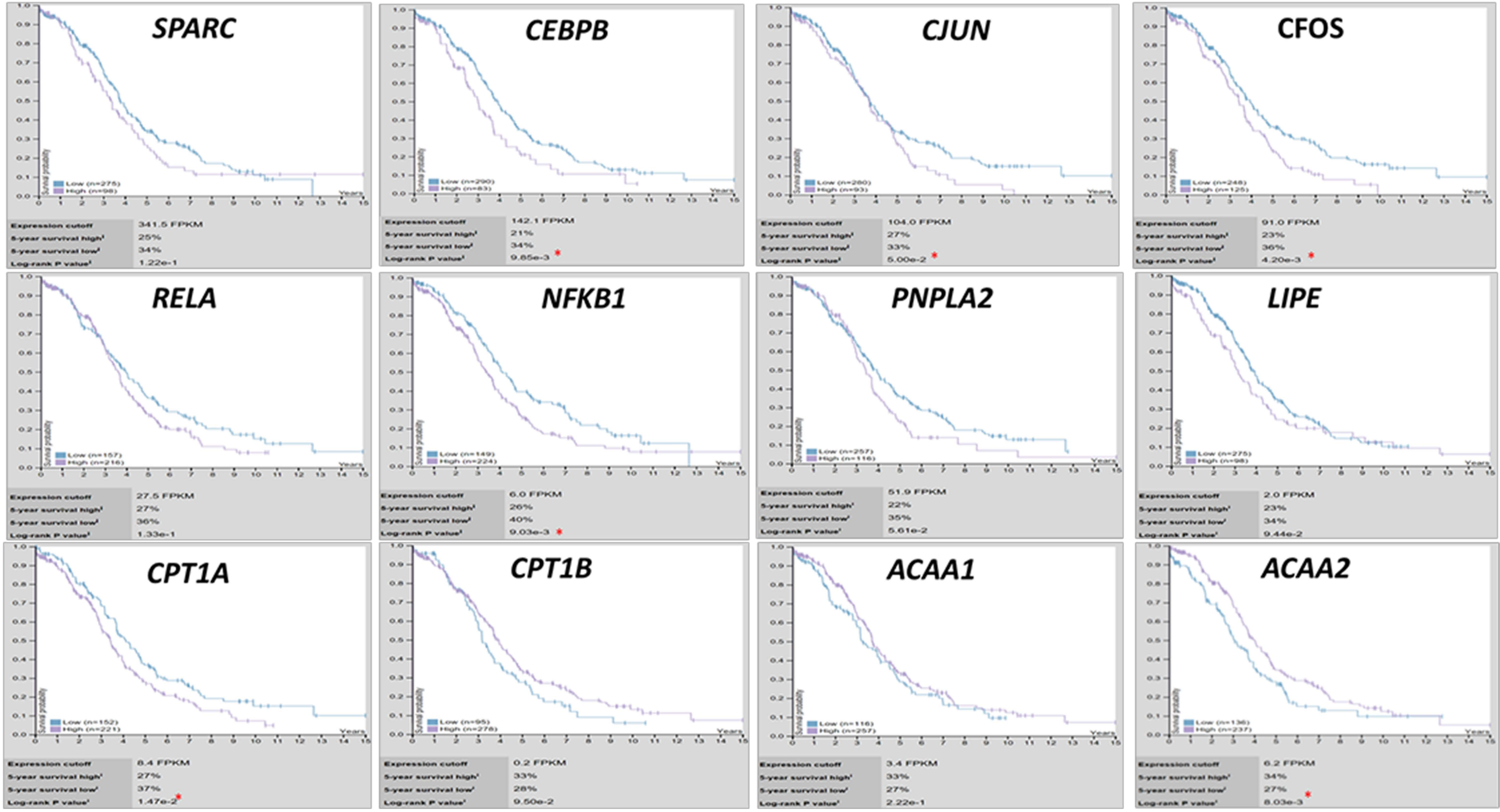

# Supplement Figure 9:

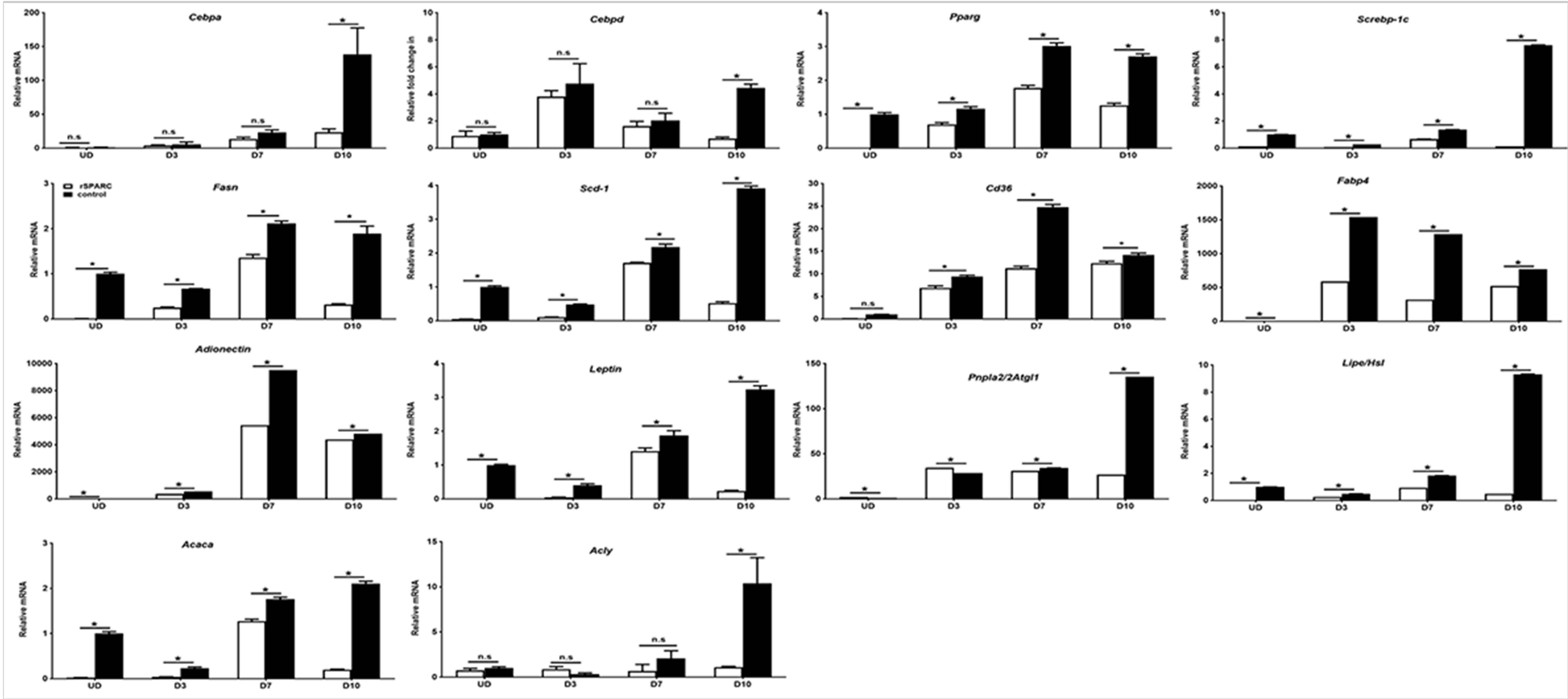

**Supplement Figure Legends:**

**Supplement Figure 1: Isolation of primary mouse preritoneal stromal cells. A.** Schema of the isolation of omental adipocytes from $SP^{-/-}$ *and* $SP^{+/+}$. **B.** Oil O red staining of freshly isolated primary adipocytes. **C.** α-smooth muscle actin (α-sma) staining showing fibroblastoid phenotype of omental stromal cells. **D.** Western blot showing the expression of SPARC protein in $SP^{-/-}$ *and* $SP^{+/+}$ adipocytes. **E.** Phase contrast (left), fluorescence (middle) and merged phase/fluorescent images of ID8-GFP cells showing the GFP expression levels. Scale bars, 100µm. **F.** Coomassie blue stained SDS-PAGE (4-20%) gel showing rhSPARC molecular weights at the indicated concentrations reconstituted in DPBS containing 0.1% BSA under reducing and non-reducing conditions. Five microliters of 0.1% BSA alongside the samples in lane 10.

**Supplement Figure 2: Homing of Ovarian Cancer cells towards normal adipocytes is mediated through secreted factors. A.** Bar graphs of the means ± SEM of the levels of cytokines and chemokines secreted in CM of normal $SP^{+/+}$ and $SP^{-/-}$ adipocytes, *$p$<0.05 comparing $SP^{+/+}$ and $SP^{-/-}$ adipocytes, Student's *t*-test. **B.** Bar graphs of the means ± SEM of the relative fluorescence intensity of ID8-GFP that homed to $SP^{+/+}$ and $SP^{-/-}$ *in vivo* in the presence of functional blocking antibodies to the indicated chemokines and their isotype controls. *$p$<0.05 comparing $SP^{+/+}$ and $SP^{-/-}$ within each condition; $^{\#}p$<0.05 compared to isotype control. $^{\$}p$<0.05 comparing the $SP^{-/-}$ to the corresponding isotype control. **C.** Bar graphs of the means ± SEM of the levels of cytokines and chemokines secreted in CM of normal $SP^{+/+}$ and $SP^{-/-}$ CAA. *$p$<0.05 comparing $SP^{+/+}$ and $SP^{-/-}$ adipocytes, Student's *t*-test. **D.** Schema of the homing/migration of ID8 cells towards normal Adi and CAA and their CM. Bars report mean ± SEM of the fold increase in migration compared to control medium considered as 1. *$p$<0.05 comparing $SP^{+/+}$ and $SP^{-/-}$. **$p$<0.05 comparing normal Adi to CAA. ***$p$<0.05 comparing migration towards adipocytes to 72h CM.

**Supplement Figure 3: Adipcyte-SPARC inhibits co-culture-induced activation of cEBPβ, NFkB and AP-1 in primary human omental adipocytes and human OvCa cells A.** Schematic illustration of the experimental design of the co-cultures of OvCa cells and omental adipocytes. **B.** Primary human omental adipocytes overexpressing (hAdi-VC and hAdi-SPARC) or depleted of SPARC (hAdi-NTsh and hAdi-shSPARC) and their vector controls were transfected with luciferase reporter plasmids of cEBPβ, NFκB and AP-1 and were co-cultured with human OvCa cells for further 24h. The activation of each transcription factor was determined by measuring the luciferase reporter activity in each cell type. Bars represent mean

± SEM of one of three experiments performed in triplicates. *$p<0.05$ comparing hADi-NTsh and hAdi-shSPARC **$p<0.05$ comparing hAdi-VC and hAdi-SPARC; and $^{\#}p<0.05$ student's *t*-test comparing cells in single to co-cultures; comparing OvCa cells in single to co-culture with hAdi-VC and hAdi-SPARC; Student's *t*-test **C.** Treatment of mon-and co-cultures of OvCa cells and adipocytes with rSPARC (5µg/ml). The activation of each transcription factor was determined by measuring the luciferase reporter activity in each cell type. Bars represent mean ± SEM of one of three experiments performed in triplicates. *$p<0.05$ comparing cells with/without rSPARC; $^{\#}p<0.05$ student's *t*-test comparing cells in single to co-cultures.

**Supplement Figure 4: The proliferation of the OvCa cell lines in mono and co-cultures with adipocytes** (under same experimental conditions of luciferase reporter assays **Figure 3C** and **Supplement Figure 3B-C**) was determined by CyQuant assay at the start of the experiments with mono- and co-cultures (0h) and at the end of the experiment (24h). Fold changes of the DNA content (proliferation) was determined for each cell type at 0 and 24h. Bars represent mean ± SEM of fold change in proliferation measured at 24h over 0h at the start of co-culture. *p* values were determined by unpaired Student's *t*-test. These fold changes were used to correct for the luciferase activity levels presented in **Figure 3C** and **Supplement Figure 3B-C.**

**Supplement Figure 5: Exogenous rSPARC suppresses co-culture-induced activation of cEBPβ, NFkB and AP-1 in primary human omental adipocytes and human OvCa cells. A.** the expression of the transcripts of IL-6, CCL2, Leptin, VEGF, and TNFα was determined in human OvCa cells in single and co-cultures with primary human adipocytes (hAdi) as described in **Supplement Figure 3**. Bars represent mean ± SEM of the fold changes in the transcript in ID8 cells in co-cultures compared to single cultures considered as 1 in one of three experiments performed in triplicates. **B.** Bars represent the mean ± SEM of the fold changes in the transcript levels in adipocytes in co-cultures compared to single cultures considered as 1. *$p<0.05$, unpaired Student's *t*-test.

**Supplement Figure 6: A.** H&E sections of omental metastasis of HGSC specimens at indicated magnifications. **B.** Immunofluorescence showing the expression of SPARC in tumor cells (T) and adipocytes (A).

**Supplement Figure 7: Effect of blockade of NFkB, AP-1 and cEBPβ on their activity in OvCa cells and adipocyte:** Treatment of ID8 and adipocytes in single and co-cultures with NFkB cell permeable peptides S276 pep and NLS pep inhibited NFkB and cEBP luciferase

reporters in adipocytes **A-B,** and ID8 cells**, C-D.** *$p<0.05$ between controls and inhibitor treatments, **$p<0.05$ comparing $SP^{+/+}$ and $SP^{-/-}$ adipocytes. ***$p<0.05$ comparing cells in mono- and co-cultures**. E.** Treatment of ID8 and adipocytes in single and co-cultures with JNK inhibitor SP600125 inhibited co-culture induced AP-1 luciferase reporter in both cell types. *$p<0.05$ comparing controls with inhibitor treatments. Bars represent mean s± SEM of one of 2 experiments each performed in quadruplicates. **$p<0.05$ comparing $SP^{+/+}$ and $SP^{-/-}$ adipocytes. $^{\$}p<0.05$ comparing adipocytes in mono- and co-cultures with ID8 cells, $^{\#}p<0.05$ comparing ID8 in mono can co-cultures with adipocytes; unpaired Student's *t*-test. **F.** The proliferation of the ID8 cells in mono and co-cultures with $SP^{+/+}$ and $SP^{-/-}$ adipocytes (under same experimental conditions of luciferase reporter assays **in A-E** was determined by CyQuant assay at the start of the experiments with mono- and co-cultures (0h) and at the end of the experiment (24h). Fold changes of the DNA content (proliferation) was determined for each cell type at 0 and 24h. Bars represent mean ± SEM of fold change in proliferation measured at 24h over 0h at the start of co-culture. *$p<0.05$ compared to control ID8 cells in mono-cultures. #p<0.05, comparing ID8 cells in coculture with $SP^{+/+}$ and $SP^{-/-}$ adipocytes, unpaired Student's *t*-test. These fold changes were used to correct for the luciferase activity levels presented in **A-E.**

**Supplement Figure 8:** Inhibition of NFkB (S276 and NLS peptides) and AP-1 (JNK inhibitor, SP6000125) inhibited co-culture-induced FFA release from adipocytes in mono and co-cultures with ID8 cells. Bars represent means ± SEM fluorescent intensity of the released of FFA from a representative of 3 independent experiments each preformed in quadruplicates. *$p<0.05$ comparing controls with inhibitor treatments, **$p<0.05$ comparing $SP^{+/+}$ and $SP^{-/-}$ adipocytes, ***$p<0.05$ comparing mono- and co-cultures; Student's *t*-test.

**Supplement Figure 9: Effect of SPARC on adipogenic differentiation factors in 3T3L1 adipocytes.** 3T3L pre-adipocytes were stimulated to differentiate into adipocytes the presence with the appropriate media as described in "Material and Methods" in the presence or absence of 5µg/ml recombinant murine SPARC. mRNA was isolated at the indicated time points. Bars report means ± SEM of the expression levels of the indicated genes of a representative of 2 experiments each performed in triplicates. *$p<0.05$, Student's *t*-test.

**Supplement Figure 10:** Bars depict means ± SEM of lipid metabolites measured in ID8 omental tumor nodules isolated 6 weeks after ip injection of ID8 in $SP^{+/+}$ and $SP^{-/-}$ mice (n=6/genotype). $p<0.05$, Student's *t*-test.

**Supplement Figure 11:** Kaplan Meier curves curated from TCGA and Protein Atlas (https://www.proteinatlas.org/) showing the correlation of the expression of cEBPβ, AP-1 (cJun and cFos) and NFκB (RelA, and NFκB1) and downstream target inflammatory and metabolic genes with patients' survival. *$p$<0.05.

**Supplement Figure 1: Patients' data of tumor tissue specimens.**

**A. WF-TTPSR (High Grade Serous):**

| | **pT1+pT2** | **pT3+** |
|---|---|---|
| **Number** | 6 | 29 |
| **Age** | 61.5 ± 4.66 | 65.17 ± 2.113 |
| **Grade**<br>**G1+G2**<br>**HG (G3+G4)** | <br>1<br>5 | <br>1<br>28 |

**B. CHTN (serous papillary and poorly differentiated):**

| | **pT1+pT2** | **pT3+** |
|---|---|---|
| Number | 10 | 30 |
| Age | 65.7 ± 5.916 | 63.2 ± 2.288 |
| Grade:<br>G1+G2<br>HG (G3+G4) | <br>5<br>5 | <br>15<br>15 |

**Supplement Table 2: Antibodies**

| **Primary and secondary Antibodies** | **Catalog #** | **Dilution** | **Molecular weight** |
|---|---|---|---|
| Rabbit Anti- SPARC (CST) | 8725 | 1:250 (40 ug) WB<br>1:100 IHC (Paraffin and IF) | 43 kDa |
| Mouse Anti-SPARC (Abnova) MAB0278 | MAB0278 | 1:500 WB<br>1:100 IHC (Paraffin and IF) | 43 kDa |
| Rabbit Anti- FABP4 (CST)<br>Fatty acid binding protein | 3544 | 1:1500 (25 ug) WB<br>1:200 IF | 15 kDa |
| Rabbit Anti-CD36 (CST) | 14347 | 1:200 IF | |
| Rabbit Anti- CEBP Alpha (CST)<br>CCAAT/enhancer-binding proteins | 8178 | 1: 1500 (25 ug) WB | 42 kDa |
| Rabbit Anti- CEBP Beta (CST)<br>CCAAT/enhancer-binding proteins | 3084 | 1: 500 (25 ug)<br>1:200 IHC (Paraffin and IF) | 38, 41 kDa |
| Rabbit Anti- PPAR Gamma (CST)<br>Peroxisome proliferator-activated receptor | 2435 | 1: 250 (25 ug) | 53, 57 kDa |
| Rabbit Anti- HSL<br>(Hormone Sensitive Lipase)<br>(CST) | 4139 | 1:3000 (25 ug) | 81, 83 kDa |
| Rabbit- Anti- Phospho CEBP Thr 235 (CST) | 3084 | 1:500 | 19 k Da LIP; 36-38 kDa LAP |
| Rabbit Anti- ATGL<br>(CST) | 2439 | 1:500 | 54 kDa |
| Mouse Anti- tubulin (Sigma) | T5168 | 1:5000 | 55 kDa |
| Anti-rabbit and anti-mouse HRP-conjugated antibodies (ThermoFisher) | Cat # 31460<br>Cat # 31430 | 1:2000 (WB)<br>1:400 (IHC-P) | |
| NIR680 and 800 anti-mouse and anti-rabbit secondary antibodies (Licor) | [P/N 925-32210]<br>[P/N 925-32211]<br>[P/N 925-68020]<br>[P/N 925-68021] | 1:10000 (WB) | |
| Alexa-fluor 488 anti-rabbit antibodies. (Invitrogen) | Cat # A-11034 | 1:400 (IF) | |
| Alexa-fluor 594 anti-rabbit antibodies. (Invitrogen) | Cat # A-11032 | 1:400 (IF) | |

**Supplementary Table 3: List of human primers used for real-time PCR**

| **Gene** | **Forward Primer sequence [5'-3'] Oligo** | **Reverse Primer sequence [5'-3'] Oligo** |
|---|---|---|
| GAPDH | AGGGCTGCTTTTAACTCTGGT | CCCCACTTGATTTTGGAGGGA |
| *IL6* | GGTACATCCTCGACGGCATCT | GTCCCTCTTTGCTGCTTTCAC |
| *CCL2* | CAGCCAGATGCAATCAATGC | GCACTGAGATCTTCCTATTGGTGAA |
| *VEGFA* | CTACCTCCACCATGCCAAGT | GCAGTAGCTGCGCTGATAGA |
| *TNFA* | GGAGAAGGGTGACCGACTCA | CTGCCCAGACTCGGCAA |
| *Leptin* | GAAGACCACATCCACACACG | AGCTCAGCCAGACCCATCTA |

**Supplementary Table 4: List of mouse primers used for real-time PCR**

| Gene | Forward Primer sequence [5'-3'] Oligo | Reverse Primer sequence [5'-3'] Oligo |
|---|---|---|
| *18S rRNA* | GTAACCCGTTGAACCCCATT | CCATCCAATCGGTAGTAGCG |
| *Cd36* | CCTTAAAGGAATCCCCGTGT | TGCATTTGCCAATGTCTAGC |
| *Gapdh* | CATTGTGGAAGGGCTCATGA | TCTTCTGGGTGGCAGTGATG |
| *Il-6* | AAAGAGTTGTGCAATGGCAATTCT | AAGTGCATCATCGTTGTTCATACA |
| *Ccl2* | GTTGGCTCAGCCAGATGCA | AGCCTACTCATTGGGATCATCTTG |
| *Vegfa* | CCTCCTCAGGGTTTCGGGAACCA | ACCCAAAGTGCTCCTCGAAGGATC |
| *Tnfa* | CATCTTCTCAAAATTCGAGTGACAA | TGGGAGTAGACAAGGTACAACCC |
| *Fabp4* | AAGGTGAAGAGCATCATAACCC | TCACGCCTTTCATAACACATTCC |
| *Pparg* | GGAAGACCACTCGCATTCCTT | GTAATCAGCAACCATTGGGTCA |
| *Srebp-1c* | GATCAAAGAGGAGCCAGTGC | TAGATGGTGGCTGCTGAGTG |
| *Fasn* | AGAGATCCCGAGACGCTTCT | GCCTGGTAGGCATTCTGTAGT |
| *Scd1* | TTCTTACACGACCACCACCA | CCGAAGAGGCAGGTGTAGAG |
| *Hsl/Lipe* | TCTATGCGCAGGAGTGTGTC | TTGACATCAGAGGGTGTGGA |
| *Atgl* | CAACGCCACTCACATCTACGG | TCACCAGGTTGAAGGAGGGAT |
| *Acaca* | CTCCAGGACAGCACAGATCA | GCCGAAACATCTCTGGGATA |
| *Leptin* | CTCATGCCAGCACTCAAAAA | AGCACCACAAAACCTGATCC |
| *Adiponectin* | AGACCTGGCCACTTTCTCCT | ATCCAACCTGCACAAGTTCC |
| *Cebpa* | CAAGAACAGCAACGAGTACCG | GTCACTGGTCAACTCCAGCAC |
| *Cebpb* | ATCGACTTCAGCCCCTACCT | GGCTCACGTAACCGTAGTCG |
| *Cebpd* | CGACTTCAGCGCCTACATTGA | GAAGAGGTCGGCGAAGAGTT |
| *Acly* | CAGCCAAGGCAATTTCAGAGC | CTCGACGTTTGATTAACTGGTCT |
| *Acat1* | CTGGGCGCAGGTTTACCTAT | GGTGTTGCTCCTCTGCTCAT |
| *Acat2* | ATTGTTGAAAGGTGGGCAGC | GGTAACATCCCATCCCGTCA |
| *Hadh* | TCGTGAACCGACTCTTGGTG | TCTTCCTTAGACGCATCGCC |
| *Crot* | ATTGGCTGGAAGAGTGGTGG | GAGTCCCTTCCTTTGGAGGC |
| *Acaa2* | GGGGCCTTCTCAAGGACTTC | ACATTGCCCACGATGACACT |
| *Acaa1* | AGGCCCTCTAAAGACCCCAT | GGTGGGTCCTACCTACTCGT |
| *Cpt1a* | GGACTCCGCTCGCTCATT | GAGATCGATGCCATCAGGGG |
| *Cpt1b* | CCTGGGATGCGTGTAGTGTT | CCTGGGATGCGTGTAGTGTT |
| *Slc25a20* | CATGTGCCTGGTGTTTGTGG | GGTGGCTGTCCAGACAAACT |
